# Behavioral Constraint Legibility Under Concealment

**DOI:** 10.5334/joc.515

**Published:** 2026-08-19

**Authors:** Assaf Benchetrit, Liliya Benchetrit

**Affiliations:** 1 University of New Hampshire, Paul Creative Arts Center, Theatre & Dance Department, Durham, NH, USA; 2 Massachusetts Eye and Ear, Harvard Medical School, Boston, MA, USA

**Keywords:** Action and perception, Stimulus development, Embodied cognition, Event cognition

## Abstract

Everyday cognition requires observers to find organization in continuous behavior as surface form changes. Behavioral streams can carry a constraint that governs what remains coherent across variation. This study examines whether such a constraint can remain publicly legible when it structures an action stream and remains concealed from observers.

The study examined this question in *Untitled, Literally*, a full-length abstract choreographic work presented in five public performances. Eight sections were constructed with Essence-to-Movement (EtoM), a procedure for preserving a specified behavioral constraint across varied movement conditions. One section, *Drink Me*, served as an internal contrast. Following the performances, audience members completed an anonymous voluntary questionnaire (N = 393).

We tested the hypothesis that observers who reported structural routes into the work would show lower uncertainty, stronger active meaning-making, and lower preference for the internal contrast. The observed profile was consistent with this hypothesis. Structural-entry reports were associated with lower uncertainty, stronger active meaning-making, and sharper within-work discrimination between constraint-governed material and the internal contrast. These associations are interpreted within a post-performance self-report design under concealment.

## 1. Introduction

### 1.1 Perceiving organization in continuous behavior

Everyday cognition depends on extracting organization from continuous streams of behavior (Barker, 1963; Newtson, 1973). Observers segment unfolding activity into events, track agents and objects across change, and form expectations about what can happen next (Zacks & Tversky, 2001; Zacks et al., 2007; Kurby & Zacks, 2008; Radvansky & Zacks, 2017).

Live multi-agent movement intensifies this problem. Bodies, timing, spacing, force, and relation can change while an underlying organization remains available to observers. This study turns that cognitive challenge into an experimental design problem: a behavioral constraint is specified during construction, concealed from observers, and evaluated through post-performance reports after exposure to the realized stream.

Several cognitive traditions locate organization in changing activity. Ecological work emphasizes information in organism-environment relations, including affordances, invariants, kinematic information, and coupled dynamics (Gibson, 1979; Runeson & Frykholm, 1983; Warren, 2006). Enactive accounts emphasize embodied action and sense-making in organism-environment coupling (Varela et al., 1991; Thompson, 2007; De Jaegher & Di Paolo, 2007; Di Paolo et al., 2017). Predictive-processing and related free-energy accounts describe perception and action as expectation-guided updating in which prediction error, precision, and uncertainty have explicit theoretical roles (Clark, 2013; Friston, 2010). Event-cognition accounts connect continuous activity to segmentation and event-model updating (Zacks et al., 2007; Radvansky & Zacks, 2017).

Dance spectatorship and dance cognition provide a relevant comparison field for the present study. Dance supplies the live performance context; constraint legibility remains the broader methodological object. Live movement unfolds as an interpretable event, and audience uptake depends on both performance features and viewer-side resources. Empirical work on contemporary dance shows that responses vary with expressivity, liveness, and audience characteristics in particular works and viewing contexts (Christensen et al., 2025). Engagement and confusion can also coexist in contemporary dance viewing, with engagement related to prior dance experience rather than liveness alone (Lee et al., 2025). At the level of sequential organization, novice observers can acquire implicit sensitivity to regularities governing unfamiliar dance-movement sequences after exposure (Opacic et al., 2009), and live dance has been studied as a temporally unfolding audience-response field using continuous measures during performance (Stevens et al., 2009). Dance spectatorship is also multimodal: sound-movement context can change what becomes salient in the experience of movement (Reason et al., 2016), and qualitative audience research describes kinesthetic and empathetic modes of response to watching dance (Reason & Reynolds, 2010). Together, these sources support using dance as a high-variance behavioral field for studying organization in live, continuous action.

The present study asks what organization remains available when the construction rule that generated a behavioral stream remains concealed. The contribution is methodological: the article specifies a behavioral constraint before construction, uses that constraint to govern a continuous movement field, conceals the construction rule from observers, and evaluates whether post-performance reports show an audience-level legibility profile under concealment.

### 1.2 A behavioral-constraint design principle

A behavioral-constraint design treats a specified organizing constraint as the primary experimental object. In this article, law names that constraint: the relation that determines which continuations remain inside the same organized family as the behavioral stream changes. Construction begins by specifying what later material must preserve to belong to the same stream. Construction can remain open at the level of movement generation, while the condition of belonging is fixed prospectively: candidate material is retained only when it preserves the declared constraint.

In cognitive terms, observers recover organization in continuous streams by tracking stable relational structure across changes of surface form. Event-perception research formalizes this capacity in terms of segmentation, event models, and hierarchical organization of ongoing activity (Zacks & Tversky, 2001; Zacks et al., 2007; Kurby & Zacks, 2008; Radvansky & Zacks, 2017).

The specified origin is denoted ϕ. The symbol marks the construction-side origin from which the operative invariant I(ϕ) is distilled. Observers encounter the realized performance and the post-performance reporting channel while ϕ remains a construction-side specification.

### 1.3 Essence-to-Movement (EtoM) architecture

A behavioral history can change in surface while remaining answerable to one governing constraint. Timing, spacing, force, role, material, task-space, or local configuration may shift, yet the same law may continue to organize what can follow. Essence-to-Movement (EtoM) is the construction architecture used here to build that continuity and to determine which realized histories remain admissible under change.

In this article, ϕ denotes the specified origin for the instantiation. EtoM(ϕ) resolves ϕ into a law-preserving construction sequence:

Essence(ϕ) → Abstract Form(ϕ) → Structured Form(ϕ) → Embodiment(ϕ)

The sequence proceeds through four construction questions:

First, which behavioral law governs continuation? This is Essence(ϕ), denoted by I(ϕ): the operative invariant distilled from ϕ.

Second, which law-bearing functions carry that invariant? This is Abstract Form(ϕ), denoted by F(ϕ): the finite operation basis that articulates the invariant into distinct functions.

Third, which rule-governed task-spaces house those functions? This is Structured Form(ϕ), denoted by V(ϕ): the vessels or task-spaces in which the functions operate under constraint.

Fourth, which realized histories are admitted through the five Continuity Layers? This is Embodiment(ϕ), denoted by R(ϕ): the set of realized histories admitted through the five Continuity Layers (CL_1_–CL_5_).

The Continuity Layers are construction-side checks that test law-preserving continuation from five directions:

- CL_1_ tests law coherence.
- CL_2_ tests endogenous drive.
- CL_3_ tests structural organization.
- CL_4_ tests surface pattern stability under ordinary variation.
- CL_5_ tests task-space coherence.

Together, these layers determine which candidate histories remain inside R(ϕ).

Admissible continuation, denoted by AC(ϕ), is the membership condition for R(ϕ). A candidate continuation enters R(ϕ) when it preserves I(ϕ), uses F(ϕ), unfolds within V(ϕ), and satisfies the Continuity Layers. Drift names the weakening of answerability to I(ϕ); breakdown marks failure of admissibility.

Expression, symbol, and thematic material enter EtoM through structural service to the governing constraint. Material is admitted when it preserves the law across transformation; surface relevance alone is insufficient.

### 1.4 Observer-side legibility under concealment

Construction fixes R(ϕ) before observer-side access begins. The observer system, denoted by O, encounters realized histories under specified observation conditions. In the present study, those conditions consist of ordinary theatrical viewing followed by an anonymous post-performance questionnaire.

Concealment, denoted by C(ϕ; O), is the observation condition under which the system-side law, the EtoM decomposition, and the admissibility rules are withheld from O. The realized performance and the post-performance questionnaire remain available, while the constraint specification is not provided as a usable instruction set.

Observer-side legibility, denoted by L(ϕ; O), is the readout relation from R(ϕ) to O. It concerns what preserved organization becomes reportable from realized histories under the stated observation condition. Legibility is evaluated through alignment among reports: under concealment, evidence for legibility is present when reports show organization consistent with the constraint-governed profile.

At a domain-agnostic level, legibility is assessed through four dimensions:

- Detectability: whether O registers behavior as organized, structured, and relationally patterned.
- Trackability: whether O maintains a stable grip on one coherent tendency across variation and changes of condition.
- Actionability: whether O can use the organization to guide expectations, coordinate actions, or evaluate what happens next.
- Discriminability: whether O distinguishes law-keeping histories within the declared working range from histories that violate I(ϕ) or abandon the law.

Equivalence of form, denoted by EF(ϕ), names structural alignment across different descriptions. Observers may produce different verbal summaries while tracking the same organizing constraint. Under concealment, that alignment is evaluated at the level of form rather than shared vocabulary.

In the present study, recognition-type reports serve as indicators of detectability and trackability, active meaning-making reports serve as indicators of actionability, and preference for the internal contrast serves as an indicator of discriminability. These indicators are interpreted as a bounded post-performance self-report profile under concealment.

**Figure 1:**
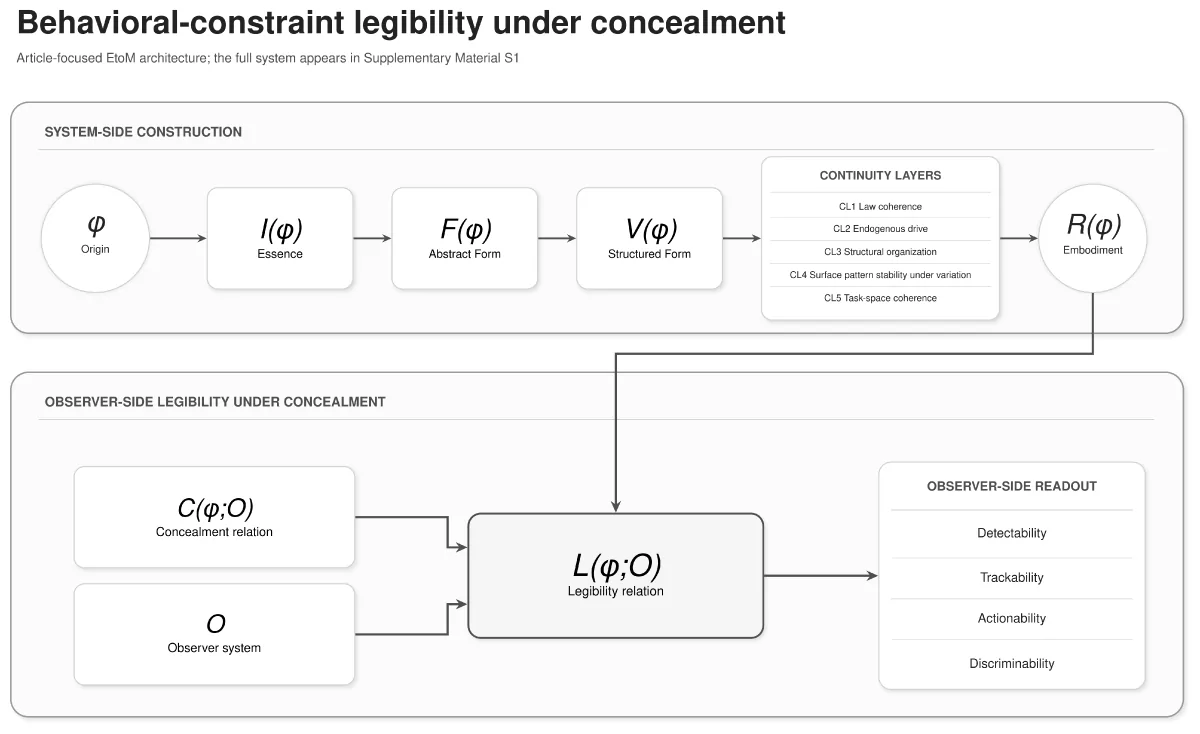
Article-focused EtoM architecture used in the present study. The figure shows the construction path from ϕ through R(ϕ), the concealment relation C(ϕ; O), the observer system O, and the legibility relation L(ϕ; O). The complete EtoM architecture appears in Supplementary Material S1.

Figure 1 summarizes the article-specific relation between the EtoM construction path, concealment, the observer system, and observer-side legibility.

### 1.5 Illustrative toy model: a cooperative ball game

A group of players keeps one shared ball in motion. They throw, catch, adjust spacing, recover from small errors, and continue playing as the arrangement changes. An observer watching from outside can see that some continuations keep the game alive, while drops, arbitrary resets, or unrelated movements break the organization.

This example gives a compact physical instantiation of EtoM. The originating frame, ϕ, is cooperative play with one shared ball. At this stage, the frame allows several possible organizing constraints: the ball must remain off the ground, each player may hold it for a fixed duration, the same partner may receive a pass after another partner has received one, or all players may keep repositioning while play continues.

For this toy model, the selected Essence is off-ground continuity. The operative invariant, I(ϕ), is that the shared ball remains off the ground while play is live. This invariant governs what counts as continuation: a throw, catch, recovery, or repositioning belongs when it keeps off-ground continuity active, whereas a play history in which the ball repeatedly comes to rest on the ground leaves the admissible range.

Abstract Form identifies the minimal functions needed to carry that invariant. In this case, the functions are: generate a catchable throw, receive and stabilize the ball, and reposition so future catches remain possible. These action capacities carry off-ground continuity under changing conditions.

Structured Form embeds those functions in task-spaces. One vessel may be a fixed pair exchange at short distance. Another may be a passing circle in which the same partner receives a pass after another partner has received one. A third may be a roaming configuration in which all players move while the ball remains live. Each vessel changes the surface conditions of play, while the same invariant constrains what can happen next.

Embodiment is the realized play history: the actual sequence of throws, catches, misses, recoveries, repositionings, and transitions between vessels. Admissible continuation appears when off-ground continuity remains active across these changes. Drift begins when the players continue moving while off-ground continuity loses control over the next action, whereas breakdown occurs when the history leaves the admissible range of the construction.

In this toy model, the five Continuity Layers make the admission test concrete:

- CL_1_ tests law coherence: off-ground continuity must continue to govern what can follow.
- CL_2_ tests endogenous drive: the next playable condition must arise from the ongoing interaction among throws, catches, recoveries, and repositioning rather than from an arbitrary external reset.
- CL_3_ tests structural organization: the players’ actions must remain coordinated as one organized play history.
- CL_4_ tests surface pattern stability under ordinary variation: off-ground continuity must persist across changes in timing, spacing, force, trajectory, and role.
- CL_5_ tests task-space coherence: the continuation must remain coherent within the selected pair, circle, or roaming configuration.

**Figure 2:**
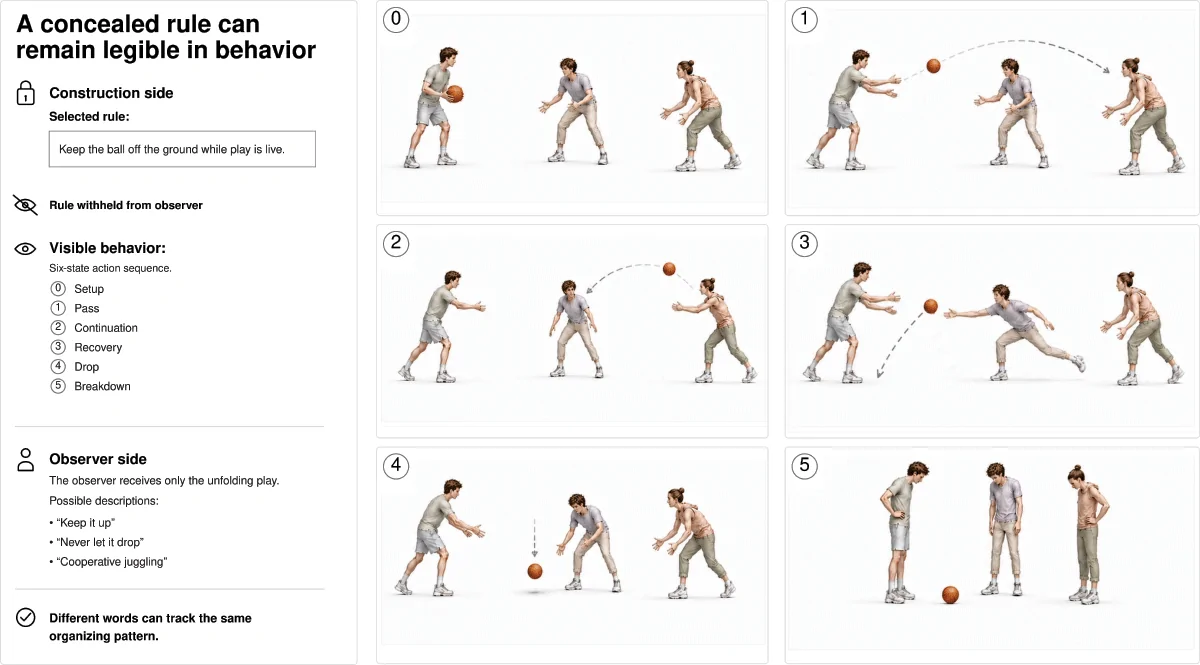
Cooperative ball-game toy model under concealment. The construction side specifies off-ground continuity and withholds that rule from the observer. The observer receives only the unfolding play and may track continuity, drift, and breakdown through their visible consequences.

Together, these checks determine whether a candidate continuation is admitted to R(ϕ). Repeated grounding, arbitrary resets, or unrelated movement fail the admission test because they no longer preserve the selected invariant through the organized play history. Figure 2 shows the visible sequence through which continuity, drift, and breakdown become available under concealment.

Under concealment, the rule remains construction-side while observers receive the unfolding play histories through visible action. Observer-side access therefore concerns what they can recover from behavior alone. Detectability appears when they register that the play is organized, and trackability appears when they follow one continuing constraint across different throws, catches, spacing changes, and role shifts. Actionability appears when they anticipate or coordinate with future play, and discriminability appears when they distinguish law-keeping histories from breakdowns, such as repeated drops or arbitrary resets.

Equivalence of form appears when different observers describe the same organization in different words. One observer may say “keep-up,” another may say “cooperative juggling,” and another may say “never let it drop.” The wording differs, while each description tracks the same organizing constraint. Under concealment, that convergence is assessed at the level of form rather than shared vocabulary.

The ball game instantiates the full EtoM construction in a minimal setting. It begins with one originating frame and one selected Essence, develops an Abstract Form, places that function set into task-spaces, realizes histories in Embodiment, admits or excludes histories through the Continuity Layers, preserves construction-side concealment, and supports observer-side access through detectability, trackability, actionability, and discriminability. It also shows how different descriptions can align through equivalence of form. Supplementary Material S1 provides the formal EtoM definitions and the full formal specification using the notation introduced above.

### 1.6 The present deployment

This paper reports a field deployment of EtoM in the choreographic work *Untitled, Literally*, a full-length abstract work. The work-level origin, ϕ_W_, was the individual-collective relation. Eight sections were constructed to preserve the operative invariant I(ϕ_W_) = individual-collective coupling under constraint. One section, titled *Drink Me*, served as an internal contrast within the same theatrical event.

Movement II, Binding-tension, provides the main construction example in the Methods section and shows how one movement-level constraint was specified, carried through task-spaces, filtered through the five Continuity Layers, and realized as performance material, while the empirical readout remains audience-level.

The central hypothesis predicted a directional profile: the Q10 structural-entry group would show a lower rate of Q6 uncertainty, a higher rate of Q8 active meaning-making, and a lower rate of Q9 preference for *Drink Me*, the internal contrast. In addition to this hypothesis, the analysis reports two descriptive questions: how strongly the audience reported engagement and recognition overall, and how that profile varied across age, dance/performing-arts experience, and familiarity with abstract performance.

## 2. Methods

### 2.1 Ethics, transparency, and language models

The study was performed in accordance with the Declaration of Helsinki and received approval from the Institutional Review Board (IRB) of a public U.S. university as minimal-risk human-subjects research. IRB-approved consent/assent procedures were used, including assent/parental permission for minors where applicable. Participation was voluntary and anonymous; no identifying, physiological, biometric, IP-address, or location data were collected. Data were stored on a secure institution-approved server. Large language models were used only for author-supervised copy-editing; study design, hypothesis specification, data analysis, and interpretation were author-conducted.

### 2.2 Setting and participants

Data were collected via a post-performance audience survey during five full-length performances of *Untitled, Literally*, a nine-section evening-length mixed ballet/contemporary work, at a U.S. university in February 2025. Performances were open to the general public. No incentives were offered. The analytic sample comprised N = 393 respondents obtained via a venue-based convenience sample across the five performances at this venue. The sampling objective was maximal coverage of attendees at a single site; targeted demographic balancing was outside the recruitment design.

### 2.3 Procedure and instrument

Prior to each performance, audience members received a brief announcement indicating that an optional post-performance survey would be available. The announcement was limited to participation logistics and maintained an attention-neutral framing with respect to structure, law, or patterns in the performance.

At the end of each performance, audience members could access an online survey on their personal devices. The opening page presented an IRB-approved information sheet describing voluntary, anonymous participation; proceeding to the questionnaire constituted consent. Participation was voluntary and uncompensated. Audience members initiated survey access themselves. The survey items provided reporting prompts, and each item was optional.

The instrument (Q1–Q11) comprised five domains:

- Background (Q1–Q3): age; self-reported dance/performing-arts experience; familiarity with abstract or unconventional performance.
- Recognition (Q4–Q5): conceptual recognition via theme selection (Q4); affective recognition via emotion selection (Q5).
- Engagement, resonance, active meaning-making (Q6–Q8): engagement mode (Q6); personal resonance with the abstract presentation of characters/voices (Q7); perceived role in meaning-making (Q8).
- Within-work discriminability (Q9): section preference under an internal contrast.
- Structural entry and language traces (Q10–Q11): structural-description endorsement (Q10); open-ended Essence prompt (Q11).

The full questionnaire (Q1–Q11), including item wording and response formats, is provided in Supplementary Material S2.

Item-level endpoints and coding-relevant roles in the EtoM/observer-legibility design (Q1–Q11):

- Q1 (Age): Characterizes audience heterogeneity and supports sensitivity checks for engagement across age strata.
- Q2 (Dance/performing-arts experience): Indexes domain experience and tests whether engagement patterns follow a simple expertise gradient.
- Q3 (Familiarity with abstract performance): Indexes prior exposure to abstract or unconventional performance.
- Q6 (Engagement mode; single-select with “I’m not sure”): The “I’m not sure” endpoint operationalizes the uncertainty marker used in the main analyses.
- Q7 (Personal resonance; single-select): The endpoint “I didn’t find any connection…” was flagged as a low-resonance marker.
- Q8 (Active meaning-making; single-select): The endpoint “I observed without feeling involved…” was flagged as a low active meaning-making marker.
- Q9 (Section preference; single-select): Selecting “*Drink Me*” is coded as preference for the unconstrained internal contrast.
- Q10 (Structural channels; multi-select): Endorsement marks a reported structural-entry route while remaining separate from construction-side constraint, EtoM decomposition, and admissibility rules.
- Q11 (Essence prompt; open response): Q11 supplies descriptive language traces under concealment; Q1–Q10 carry the inferential tests.

### 2.4 Choreographic construction and EtoM instantiation in *Untitled, Literally*

In this field study, the choreographer used EtoM to build eight sections of *Untitled, Literally* from the specified work-level constraint. The audience encountered the staged work under ordinary theatrical conditions while the construction rule remained construction-side.

**Figure 3:**
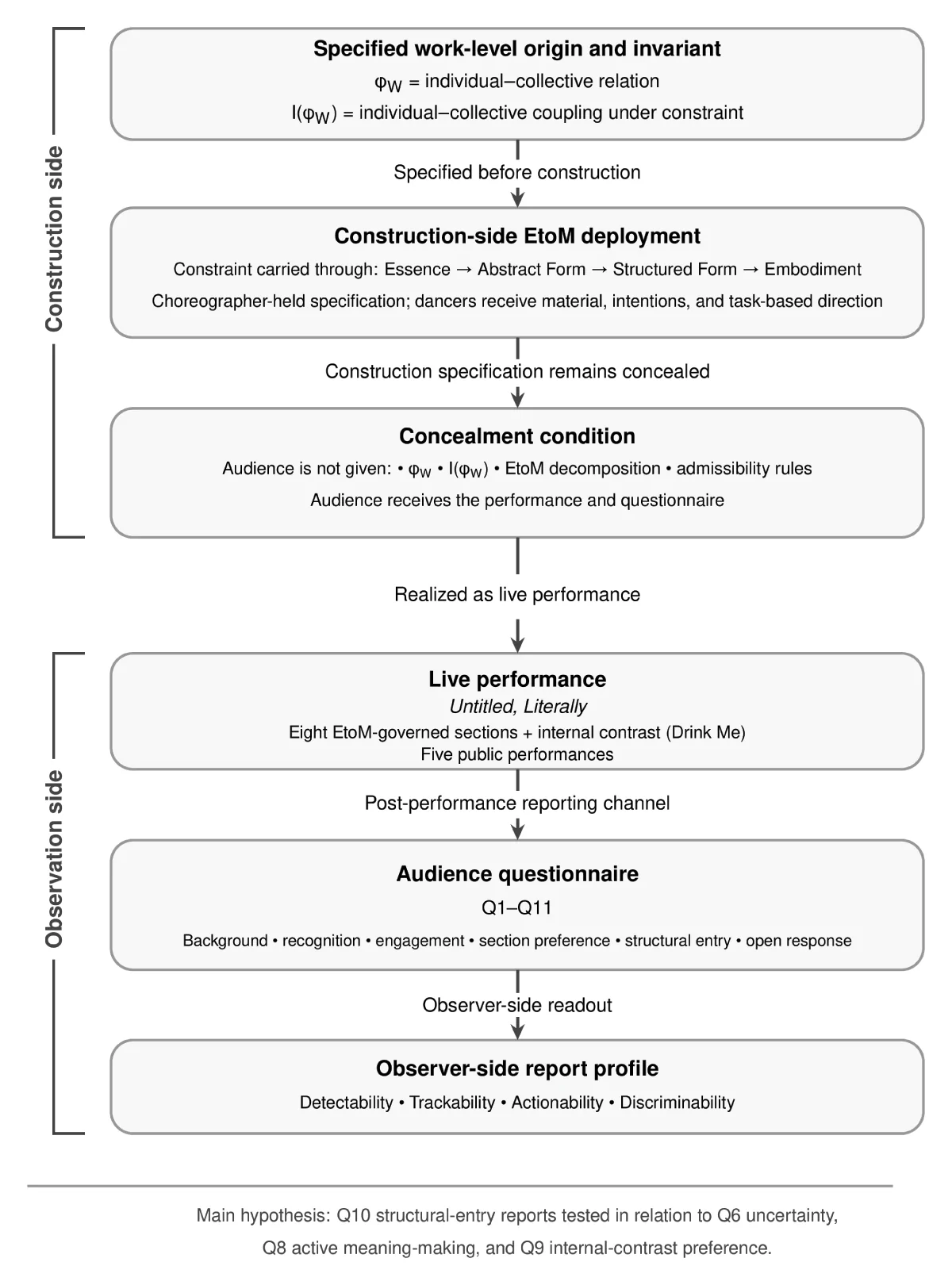
Construction-to-observation Chain for Behavioral Constraint Legibility Under Concealment.

Figure 3 summarizes the construction-to-observation chain used in this deployment.

The choreographer alone held the EtoM specification, ϕ, CL_1_–CL_5_, and admissibility rules. Dancers received movement material, performance intentions, and task-based direction through choreographic rehearsal; they were not taught EtoM as a conceptual framework or asked to apply it conceptually.

During rehearsal, construction-side control operated through choreographic specification and rehearsal selection. Material remained in the eight sections when it carried the specified relation in performance. Spacing, timing, casting, musical alignment, and theatrical shape were organized under that requirement.

All performances used the same recorded soundscape. The soundscape functioned as a fixed component of the performance context across performances, providing temporal and atmospheric context. Its contribution was not manipulated independently in the present study.

#### 2.4.1 Instantiating O in the present study

In the present deployment, O is the audience watching the live performance and reporting through the post-performance questionnaire. In the general framework, O denotes an observer system coupled to realized behavior.

Audience members received the performance and the questionnaire. They did not receive the construction-side constraint, EtoM decomposition, admissibility rules, or additional verbal or written instructions about structure, law, or pattern beyond the survey instrument.

R(ϕ) denotes the realized movement histories admitted by CL_1_–CL_5_. Observer-side legibility is evaluated through post-performance self-report measures under this concealed observation channel.

#### 2.4.2 Task-space families in *Untitled, Literally*

The larger work used five construction-side task-space families. A task-space means a practical movement situation: the bodies, objects, supports, timing conditions, and allowable actions through which the constraint can operate. The labels organized construction records rather than audience-facing survey prompts.

- Symbolic task-spaces assign the relation to stable objects or images.
- Metaphoric task-spaces realize the relation through analogical movement conditions.
- Conceptual task-spaces implement the relation as an explicit system of admissible moves.
- Progressive task-spaces distribute the availability of the relation over time.
- Subtextual task-spaces constrain overt action by limiting what can occur within the performed situation.

#### 2.4.3 Survey alignment with structural entry routes (Q10)

Q10 supplied the audience-facing structural-entry partition used later in the analysis. Its options sampled broad routes into organization suitable for a heterogeneous audience. The partition remained separate from the construction records, which contained the constraint, task-space decomposition, and admissibility rules.

#### 2.4.4 Work-level stage origin and movement-level regimes

The originating frame of the work was the individual-collective relation. For EtoM construction, this was specified as the work-level origin ϕ_W_, with the operative invariant I(ϕ_W_) = individual-collective coupling under constraint. Each EtoM-governed movement carried that relation in a different movement situation, changing the bodies, objects, coordination structures, and surface features through which the relation appeared.

Movement-level regimes name the section-level ways the shared work-level constraint is carried in performance. The labels below orient the section structure; they were not audience-facing keys or separate studies:

II: Binding–tension (EtoM construction example; specified in Section 2.5)

III: Self-location

IV: Rule gating

V: Crowd calibration

VI: Norm volatility

VII: Dominance maintenance

VIII: Command consolidation

IX: Constraint release

Movement II serves as the detailed within-work example showing how the overall EtoM construction becomes performed movement. The other labels orient the section structure while the hypothesis and outcome measures remain audience-level. Table 1 summarizes the construct-to-measure alignment used in the analysis.

**Table 1:** Construct → measure → expected direction (support pattern).


CONSTRUCT (LEGIBILITY DIMENSION)	MEASURE(S) IN THIS STUDY	EXPECTED DIRECTION (SUPPORT PATTERN)

Detectability/Trackability (observer-side contact with structured unfolding)	Q4; Q5; Q6	Higher reportable recognition in Q4–Q5 and lower uncertainty in Q6 align with stronger detectability and trackability under the stated observation channel.

Actionability/Active meaning-making	Q7; Q8	Higher resonance and stronger active meaning-making align with stronger actionability under the stated observation channel.

Discriminability	Q9	Q9 preference patterns covary with the other legibility measures in the direction specified by the observer-side profile, interpreted as within-work discriminability under a fixed order rather than as a counterbalanced causal estimate.

Structural entry routes	Q10	Higher Q10 endorsement aligns with stronger reportable structure pickup and covaries with lower uncertainty (Q6) and stronger active meaning-making markers (Q8).

Language traces and equivalence of form	Q11	Q11 responses provide descriptive traces of observer language under concealment. Q1–Q10 carry the inferential tests.


### 2.5 Movement II construction example: Binding-tension

Movement II, Binding-tension, provides the worked EtoM construction example for the Methods section. It shows how one movement-level origin is specified, resolved into an invariant, articulated as law-bearing functions, embedded in task-spaces, and realized as performance material through the five Continuity Layers. Observer-side uptake remains evaluated at the work level through the post-performance questionnaire under concealment.

The movement-level origin is denoted ϕ_II_. For this movement, the operative invariant, I(ϕ_II_), is binding-tension: one internal bind held under load across bodies, materials, and objects. Candidate material belongs when effort changes what another body, material, or object can do next while preserving that bind.

Abstract Form(ϕ_II_) fixed three law-bearing functions: (f_1_) opposed pulls seek coherence; (f_2_) continuity under variation; and (f_3_) evidence over expectation. These functions formed the closed operation basis for the movement. Later construction scheduled and recombined f_1_–f_3_ while keeping that operation basis fixed.

Structured Form(ϕ_II_) embedded those functions in three task-spaces: Fabric, Mutations, and Walker. Fabric operated as a conceptual vessel foregrounding f_1_: opposed pulls had to organize into one coupled field mediated by the cloth. Mutations operated as a metaphoric vessel foregrounding f_2_: split–recombine transitions had to preserve one traceable bind-under-load line across changing linkage structures. Walker operated as a symbolic vessel foregrounding f_3_: transmitted load had to produce mechanical consequence through the walker frame.

Embodiment(ϕ_II_) consisted of realized histories admitted through CL_1_–CL_5_. In Fabric, admissible continuation required the cloth to mediate a shared bind, with local drift repaired inside the elastic field. In Mutations, admissible continuation required recomposition to preserve one traceable bind-under-load relation across changing supports. In Walker, admissible continuation required visible effort to remain coupled to mechanical consequence through the object.

Ordinary performance perturbations included cast substitution, spacing drift, tempo variance, and minor staging noise. These variations remained inside the working range when continuity was restored within the active vessel while preserving CL_1_–CL_5_. Disturbances requiring suspension, reset, major prop failure, injury stoppage, or a change of task-space fell outside the admissible range for this construction record.

**Figure 4:**
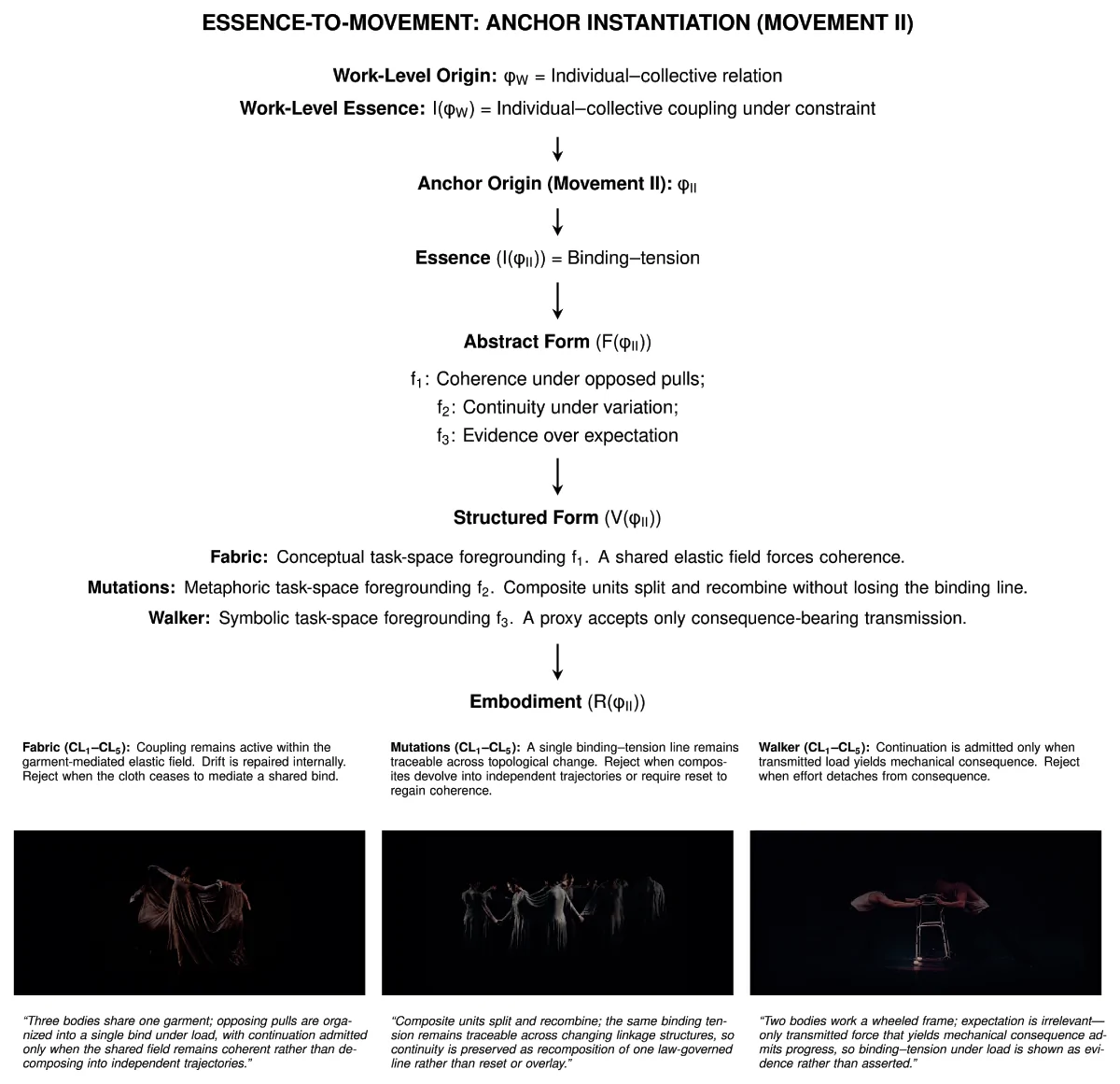
Movement II construction example: binding-tension.

Figure 4 orients this EtoM construction example. Audience uptake remains evaluated through the post-performance questionnaire under concealment.

### 2.6 Data preparation and coding

Duplicate submissions were removed using conservative timestamp-based and response-pattern checks. The retained analytic dataset contains complete Q1–Q10 records for 393 respondents. Open-ended Q11 responses were handled separately as descriptive material.

- Recognition (Q4–Q5): conceptual and affective contact under concealment. For both Q4 (Themes/ideas) and Q5 (Emotions), responses were coded as recognized when the respondent endorsed at least one substantive option (any of the pre-specified bins and/or “Other”). Responses were coded as not recognized when the respondent selected the explicit non-recognition option as the sole endorsement (for Q4: “None of these themes reflected my experience”; for Q5: “Did not reflect my experience”). When respondents selected the explicit non-recognition option together with one or more substantive options, coding followed the substantive options. This coding distinguishes explicit non-recognition from off-bin uptake while preserving “Other” as evidence of organized reporting under a constrained response scaffold.
- Engagement, resonance, and active meaning-making endpoints (Q6–Q8): Q6 (Engagement): Responses 1–5 were coded as Engaged; response 6 (“I’m not sure”) was coded as Uncertain. Q7 (Resonance): The endpoint “I didn’t find any connection…” was flagged as a low-resonance marker. Q8 (Active meaning-making): The endpoint “I observed without feeling involved…” was flagged as a low active meaning-making marker.
- Internal contrast outcome (Q9): Q9 (Section preference): Selecting “*Drink Me*” was coded as preference for the unconstrained internal contrast; selecting any other section was coded as preference for an EtoM-governed section.
- Structural-channel recognition (Q10): Q10 responses were coded as Yes when any listed structural descriptor option was endorsed. Responses were coded as No when the respondent endorsed “None of these elements resonated with me” and no structural-descriptor options.
- Open-ended traces (Q11): Q11 responses were curated as short, non-identifying traces and grouped into descriptive trace banks for qualitative presentation. Q11 characterizes the range of observer-produced language under concealment. Q1–Q10 carry the inferential tests.

### 2.7 Statistical analysis

Descriptive results are reported as counts and percentages, with Wilson 95% confidence intervals where applicable (Wilson, 1927). Associations between engagement and background variables (Q1–Q3 × Q6) are reported as sensitivity context. Detailed age categories contain sparse cells, so age is treated descriptively and checked with a collapsed under-25 versus 25-and-older Fisher exact test, excluding the undisclosed-age category.

Associations between Q10 structural-entry reports and outcomes Q4–Q9 are quantified with risk ratios and log-scale 95% confidence intervals, with two-sided Fisher exact tests. A 2 × 2 cell with zero count receives a 0.5 continuity correction for risk-ratio and confidence-interval computation. For the Q10 association family, p-values are interpreted with Benjamini-Hochberg false-discovery-rate correction as a sensitivity check. The three central hypothesis indicators—Q6 uncertainty, Q8 active meaning-making, and Q9 internal contrast preference—are evaluated as the focal hypothesis subset.

As a targeted sensitivity analysis for a simple expertise-gradient account, Q2 dance/performing-arts experience was cross-tabulated with Q10 structural-entry reports, Q6 uncertainty, Q8 active meaning-making, and Q9 *Drink Me* preference. Each 5 × 2 table was evaluated with a two-sided Fisher-Freeman-Halton exact test, with BH-FDR correction across the four Q2 sensitivity tests.

All statistical claims are interpreted as associative profiles from a post-performance self-report design under concealment.

## 3. Results

Analyses are based on N = 393 respondents. The retained dataset contains complete Q1–Q10 records for all respondents. Q11 is handled separately as open-ended descriptive material.

### 3.1 Attentional engagement (Q6)

Among respondents, 362/393 (92.1%; Wilson 95% CI [89.0%, 94.4%]) were Engaged and 31/393 (7.9%; Wilson 95% CI [5.6%, 11.0%]) were Uncertain (Table 2). Q6 responses 1–5 were coded as Engaged; response 6 (“I’m not sure.”) was coded as Uncertain.

**Table 2:** Attentional engagement distribution (Q6).


Q6 OUTCOME	COUNT	PERCENTAGE	WILSON 95% CI

Engaged (1–5)	362	92.1%	[89.0%, 94.4%]

Uncertain (6)	31	7.9%	[5.6%, 11.0%]

Total	393	100.0%	—


### 3.2 Demographics × engagement (Q1–Q3 × Q6)

Q6 was analyzed as Engaged (1–5) versus Uncertain (6) against age (Q1), dance/performing-arts experience (Q2), and familiarity with abstraction (Q3). Descriptive distributions are shown in Tables 3, 4, 5. Detailed age categories included sparse cells, so the detailed age table is treated descriptively. The collapsed age sensitivity check comparing respondents under 25 with respondents 25 and older, excluding the undisclosed-age category, yielded Fisher exact p = .818. Dance/performing-arts experience showed an aggregate association with engagement status, χ²(4) = 13.54, V = .19; the exact Fisher-Freeman-Halton test reported in Table 10 yielded p = .0052. The category profile was mixed across experience levels. Familiarity with abstract or unconventional performance did not show a clear aggregate association with engagement status, χ²(4) = 5.00, p = .287, V = .11.

**Table 3:** Engagement by Age Group (Q1).


AGE GROUP	COUNT	ENGAGED	UNCERTAIN	ENGAGEMENT RATE

11 and younger	1	1	0	100.0%

12–14	2	2	0	100.0%

15–17	11	8	3	72.7%

18–24	293	273	20	93.2%

25–34	9	9	0	100.0%

35–44	9	8	1	88.9%

45–54	28	25	3	89.3%

55 and older	38	35	3	92.1%

I prefer not to answer	2	1	1	50.0%

Total	393	362	31	92.1%


**Table 4:** Engagement by Dance/Performing-Arts Experience (Q2).


EXPERIENCE LEVEL	COUNT	ENGAGED	UNCERTAIN	ENGAGEMENT RATE

No Experience	100	91	9	91.0%

Beginner	130	118	12	90.8%

Intermediate	90	89	1	98.9%

Advanced	54	45	9	83.3%

Professional	19	19	0	100.0%

Total	393	362	31	92.1%


**Table 5:** Engagement by Familiarity with Abstraction (Q3).


FAMILIARITY LEVEL	COUNT	ENGAGED	UNCERTAIN	ENGAGEMENT RATE

Not familiar at all	130	116	14	89.2%

Slightly familiar	138	132	6	95.7%

Moderately familiar	72	67	5	93.1%

Very familiar	41	36	5	87.8%

Extremely familiar	12	11	1	91.7%

Total	393	362	31	92.1%


### 3.3 Recognition, resonance, and active meaning-making indices (Q4–Q8)

Thematic recognition (Q4) was 372/393 (94.7%; Wilson 95% CI [92.0%, 96.5%]) and affective recognition (Q5) was 387/393 (98.5%; Wilson 95% CI [96.7%, 99.3%]) (Table 6). Q4 and Q5 were coded as Recognized when respondents endorsed at least one substantive option, including “Other.” Non-recognition required endorsing only the explicit non-recognition option for that item. Within this coding, Q4 non-recognition was 21/393 (5.3%) and Q5 non-recognition was 6/393 (1.5%), corresponding to respondents who selected the explicit non-recognition option as the sole endorsement for that item. Endpoint rates for uncertainty, low resonance, and low active meaning-making are reported in Table 7. Endpoints were Q6 = “I’m not sure.”, Q7 = “I didn’t find any connection…”, and Q8 = “I observed without feeling involved…”.

**Table 6:** Recognition rates (Q4–Q5).


MEASURE	n/N	RATE	WILSON 95% CI

Thematic recognition (Q4)	372/393	94.7%	[92.0%, 96.5%]

Affective recognition (Q5)	387/393	98.5%	[96.7%, 99.3%]


**Table 7:** Uncertainty, resonance, and active meaning-making endpoints (Q6–Q8).


MEASURE	n/N	RATE	WILSON 95% CI

Q6 = “I’m not sure” (Uncertain)	31/393	7.9%	[5.6%, 11.0%]

Q7 = “No connection”	43/393	10.9%	[8.2%, 14.4%]

Q8 = “Observed only”	78/393	19.8%	[16.2%, 24.1%]


### 3.4 Layered endpoint profiles across recognition and engagement (Q4–Q8)

Composite profiles were constructed across Q4–Q8. The Recognition-domain endpoint profile required Q4 and Q5 both to indicate non-recognition, with at least one Q6–Q8 item outside its endpoint. The Engagement-domain endpoint profile required Q6–Q8 all to be at endpoints, with at least one Q4 or Q5 recognition item retained. The Complete endpoint profile required Q4 and Q5 non-recognition together with Q6–Q8 all at endpoints. The observed rates were 0/393 (0.00%; Wilson 95% CI [0.00%, 0.97%]) for the Recognition-domain endpoint profile, 9/393 (2.29%; Wilson 95% CI [1.21%, 4.29%]) for the Engagement-domain endpoint profile, and 1/393 (0.25%; Wilson 95% CI [0.04%, 1.43%]) for the Complete endpoint profile (Table 8).

**Table 8:** Layered endpoint profiles across recognition and engagement domains (Q4–Q8).


COMPOSITE PROFILE	n/N	RATE	WILSON 95% CI

Q4 and Q5 both non-recognition, with at least one Q6–Q8 item outside its endpoint	0/393	0.00%	[0.00%, 0.97%]

Q6–Q8 all endpoints, with at least one Q4 or Q5 recognition item retained	9/393	2.29%	[1.21%, 4.29%]

Q4–Q8 all endpoint-coded	1/393	0.25%	[0.04%, 1.43%]


### 3.5 Structural-entry reports and observer-side indicators (Q10 → Q4–Q9)

**Figure 5:**
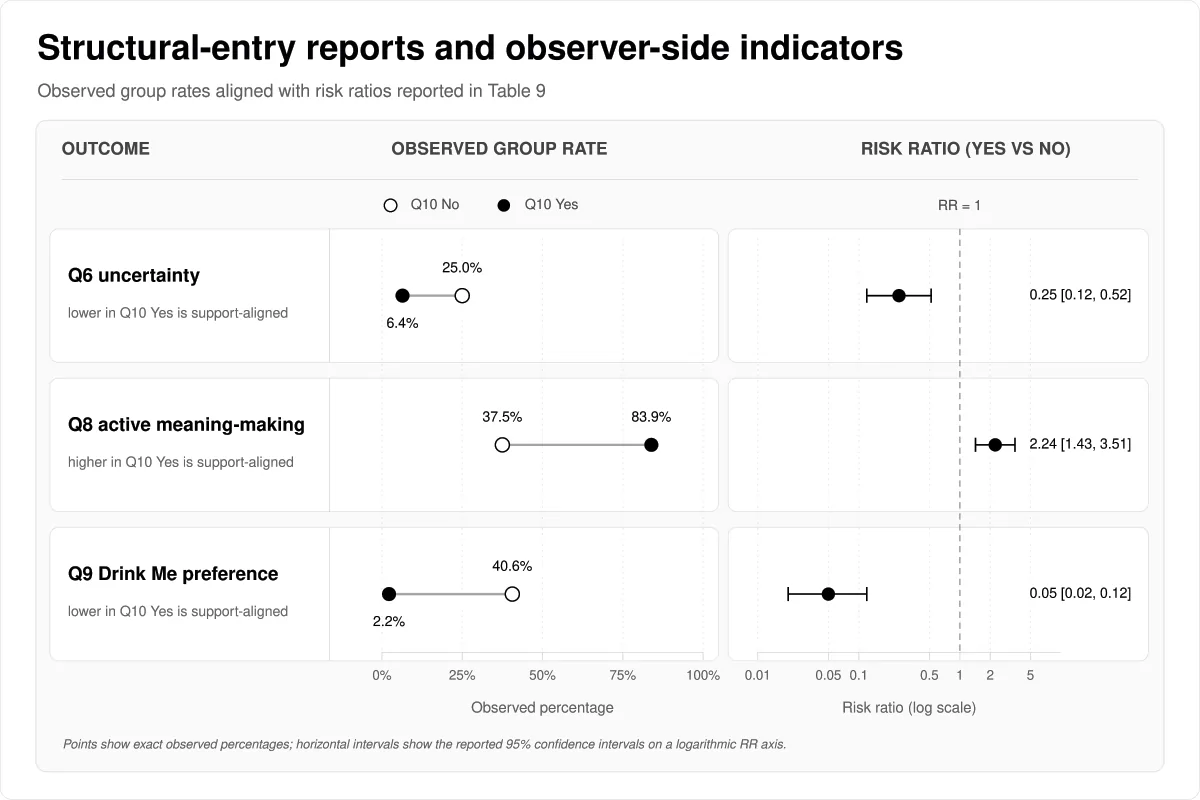
Central hypothesis indicators by structural-entry report (Q10). Percentages compare respondents who did and did not report a structural-entry route; risk ratios and 95% confidence intervals are reported in Table 9.

Q10 Yes appeared in 361/393 responses (91.9%; Wilson 95% CI [88.7%, 94.2%]); Q10 No appeared in 32/393 responses (8.1%; Wilson 95% CI [5.8%, 11.3%]). Q10 was coded Yes if any listed structural descriptor option was endorsed and No if only the explicit non-resonance option was endorsed. Associations are reported as risk ratios with 95% CIs and two-sided Fisher exact tests. BH-FDR q-values were computed across the six Q10 associations. The central hypothesis indicators were Q6 uncertainty, Q8 active meaning-making, and Q9 *Drink Me* preference. Figure 5 visualizes these central hypothesis indicators by structural-entry report.

**Table 9:** Structural-entry reports (Q10) versus recognition, engagement, resonance, active meaning-making, and section preference (Q4–Q9).


OUTCOME	Q10 = NO (n/N, %)	Q10 = YES (n/N, %)	RR (YES VS NO)	95% CI	P-VALUE (FISHER, 2-SIDED)

Q4: Thematic recognition	11/32 (34.4%)	361/361 (100.0%)	2.87	[1.80, 4.57]	3.74 × 10^–27

Q5: Affective recognition	29/32 (90.6%)	358/361 (99.2%)	1.09	[0.98, 1.22]	.0083

Q6: Uncertainty	8/32 (25.0%)	23/361 (6.4%)	0.25	[0.12, 0.52]	.0017

Q7: Personal resonance (any)	20/32 (62.5%)	330/361 (91.4%)	1.46	[1.12, 1.92]	2.95 × 10^–5

Q8: Active meaning-making (any)	12/32 (37.5%)	303/361 (83.9%)	2.24	[1.43, 3.51]	3.28 × 10^–8

Q9: Preference = “*Drink Me*”	13/32 (40.6%)	8/361 (2.2%)	0.05	[0.02, 0.12]	6.87 × 10^–11


For the six Q10 associations, BH-FDR q-values were: Q4 = 2.24 × 10^–26; Q5 = .0083; Q6 = .0020; Q7 = 4.42 × 10^–5; Q8 = 6.57 × 10^–8; Q9 = 2.06 × 10^–10.

Table 10 reports the Q2 sensitivity analysis. Q2 was not associated with Q10 structural-entry reports (p = .5841, q = .7788), Q8 active meaning-making after BH-FDR correction (p = .1036, q = .2073), or Q9 *Drink Me* preference (p = .9697, q = .9697). Q2 was associated with Q6 uncertainty (p = .0052, q = .0209). The category pattern was mixed rather than monotonic: uncertainty was 9.0% among respondents with no experience, 9.2% among beginners, 1.1% among intermediate respondents, 16.7% among advanced respondents, and 0.0% among professionals. The central profile was not reducible to a simple dance/performing-arts experience gradient.

**Table 10:** Q2 dance/performing-arts experience sensitivity analysis for central indicators.


OUTCOME	RATES BY Q2 CATEGORY (NO EXP., BEG., INT., ADV., PROF.)	FISHER P	BH-FDR Q	INTERPRETATION

Q10 structural-entry reports	94.0%, 88.5%, 92.2%, 94.4%, 94.7%	.5841	.7788	No simple expertise gradient

Q6 uncertainty	9.0%, 9.2%, 1.1%, 16.7%, 0.0%	.0052	.0209	Mixed, non-monotonic pattern

Q8 active meaning-making	73.0%, 78.5%, 87.8%, 81.5%, 89.5%	.1036	.2073	Not significant after BH-FDR

Q9 *Drink Me* preference	5.0%, 5.4%, 6.7%, 3.7%, 5.3%	.9697	.9697	No simple expertise gradient


### 3.6 Open-ended Essence responses (Q11): descriptive language traces under concealment

Q11 supplies descriptive language traces under concealment, while Q1–Q10 carry the inferential tests. Q11 responses were curated as short, non-identifying traces and grouped into thematic trace banks (Supplementary Material S3). Q11 characterizes the range of observer-produced language under concealment, including traces of pressure, tension, unity, relation, persistence, ambiguity, overload, rejection, and questioning.

## 4. Discussion

### 4.1 Behavioral-constraint design and observer-side legibility

This deployment estimates an observer-side legibility profile under concealment for one specified behavioral constraint. Audience reports showed broad contact with organization: engagement was common (Q6), and recognition items used as reporting scaffolds (Q4–Q5) approached ceiling. Those high rates characterize the reporting regime and establish a reportability floor; the main analytic leverage lies in cross-item profiles that retain variance across observers.

That leverage is carried most directly by the observer-side profile linking structural-entry reports (Q10) with uncertainty (Q6), active meaning-making (Q8), and preference for *Drink Me*, the internal contrast (Q9). In EtoM terms, the discussion question is whether reports align with a constraint-governed profile: lower uncertainty, stronger active meaning-making, and lower preference for the internal contrast among respondents who reported structural-entry routes.

### 4.2 Structural-entry reports and observer-side alignment

Q10 grouped respondents by reported structural-entry route into the work. Its options provided broad audience-facing routes into organization, including symbolism, conceptual layers, metaphor, subtext, and progression. The construction-side constraint, EtoM decomposition, and admissibility rules remained withheld. Q10 is therefore treated as a report of structural entry under the survey channel, rather than as direct identification of the system-side law.

The Results show that this grouping aligned with a broad observer-side profile. Respondents in the Q10 Yes group showed higher thematic recognition (100.0% vs. 34.4%), higher affective recognition (99.2% vs. 90.6%), lower uncertainty (6.4% vs. 25.0%), higher personal resonance (91.4% vs. 62.5%), higher active meaning-making (83.9% vs. 37.5%), and lower preference for *Drink Me*, the internal contrast (2.2% vs. 40.6%). The central hypothesis indicators were therefore carried by a profile rather than by Q10 alone: structural-entry reports aligned with lower uncertainty, stronger active meaning-making, and lower preference for the internal contrast.

This pattern is compatible with event-cognition accounts in which observers parse ongoing activity into event models tied to predictability, comprehension, and memory (Zacks et al., 2007; Kurby & Zacks, 2008), and with dance-viewing work showing that expertise can shape perceived units of movement organization (Jang & Pollick, 2011). In the present study, that expertise account was treated as a sensitivity question rather than an assumption. Dance/performing-arts experience did not show a simple expertise gradient for Q10 structural-entry reports, Q8 active meaning-making, or Q9 *Drink Me* preference; Q6 uncertainty varied by experience category, but the pattern was mixed rather than monotonic.

### 4.3 Active meaning-making and membership under constraint

Respondents who reported structural-entry routes also reported higher active meaning-making than the Q10 No group. In the Results, Q8 active meaning-making was 83.9% in the Q10 Yes group and 37.5% in the Q10 No group. The same grouping also showed lower uncertainty and lower preference for *Drink Me*, the internal contrast.

In EtoM terms, this pattern matters at the level of membership. Construction-side membership was defined by admissible continuation: realized histories belonged when they preserved the specified relation through variation. On the observer side, Q8 supplied the self-report marker for active meaning-making after exposure. Active meaning-making is therefore interpreted as part of an observer-side alignment profile, rather than as an isolated response item.

Background variables supplied sensitivity context. Dance/performing-arts experience did not show a simple expertise gradient for Q8 active meaning-making or Q9 *Drink Me* preference, and the Q6 uncertainty pattern was mixed rather than monotonic. The near-ceiling affective recognition rate (Q5 = 98.5%) provides a broad background condition for the profile. General recognition shows that the reporting channel was active; the Q10-linked profile shows where observer-side differentiation remained available within that high-recognition field.

### 4.4 Cognitive-science placement: invariants, event organization, uncertainty, and active meaning-making

The audience-facing results concern reports about organization across transformation. Ecological accounts frame perception in relation to information available in organism-environment relations, including invariants, affordances, and coupled dynamics (Gibson, 1979; Warren, 2006). Event-cognition accounts describe how observers parse continuous activity into event structures shaped by prediction, boundaries, attention, and memory (Zacks & Tversky, 2001; Zacks et al., 2007; Kurby & Zacks, 2008; Radvansky & Zacks, 2017). Within that shared question space, the present study contributes a construction-side method: a behavioral constraint is declared prospectively, carried through admissible continuations, and then evaluated through observer-side reports under concealment.

Predictive-processing and related free-energy accounts treat uncertainty, surprise, and the weighting of error signals as central to perception and action (Clark, 2013; Friston, 2010). The measurement level in the present study is post-performance self-report, rather than online prediction-error, precision-weighting, free-energy, or event-boundary measurement. Within that boundary, lower reported uncertainty is theoretically intelligible as an observer-side correlate of a more settled post-performance organization.

Enactive accounts frame cognition as active, embodied sense-making in organism-environment coupling, and participatory sense-making as a stronger claim about meaning generated in interaction itself (Varela et al., 1991; Thompson, 2007; De Jaegher & Di Paolo, 2007; Di Paolo et al., 2017). In this deployment, Q8 is used narrowly as a post-performance self-report index of active meaning-making. The technical participatory-sense-making claim is therefore held as a theoretical neighbor rather than as the measured construct.

Together, these literatures position the present findings as an observer-side profile of reported organization, lower reported uncertainty, and active meaning-making under concealment. The contribution is methodological: EtoM makes the organizing constraint explicit before construction, preserves it through admissible continuation, and then asks what remains reportable when the construction rule stays outside the observation channel.

### 4.5 Methodological contribution: the ϕ → I(ϕ) specification as a portable research unit

The ϕ → I(ϕ) specification provides a portable research unit for comparative work: the operative invariant can be held fixed while task-spaces, materials, movement vocabularies, physical substrates, or measurement interfaces vary (Hutchins, 1995). In the present deployment, I(ϕ_W_) organized multiple movement regimes within one choreographic work. Portability beyond that setting remains untested.

In this role, EtoM complements established event-segmentation and structured-action paradigms by operating upstream of observer testing. It supplies a protocol for constructing histories around a pre-specified relational constraint, which can then enter established tasks such as event-boundary marking (Monroy & Wagner, 2023) or discrimination of rule-conforming and rule-violating movement sequences (Opacic et al., 2009). Rule-governed sequence construction and controlled naturalistic stimulus design already have substantial precedent (Opacic et al., 2009; Bezdek et al., 2023). EtoM’s narrower proposal is to use explicit admission criteria to preserve one operative invariant across heterogeneous, high-variance histories. Section 4.7 specifies the independent verification and controlled comparison required to test that proposal.

### 4.6 Q11 traces and equivalence of form

After the closed-form items, respondents answered an open-ended Essence prompt in one word, phrase, or short elaboration. Supplementary Material S3 presents curated, de-identified trace banks spanning pressure, tension, unity, relation, persistence, ambiguity, overload, rejection, and questioning. These analyst-defined groupings preserve heterogeneity in observer wording and provide a descriptive language field around the quantitative profile reported in Q1–Q10.

Q11 illustrates heterogeneous observer-side encodings under concealment. Different wording can describe organization compatible with the performed field, while low-contact traces mark weaker compatibility under the observation channel. The traces do not establish direct law recovery, shared semantic interpretation, latent categories, or an independent quantitative test of equivalence of form. Q11 therefore illustrates the range of observer language, while Q4–Q10 carry the quantitative legibility profile and Q1–Q10 carry the inferential tests.

### 4.7 Limitations and Future Directions

This study estimates observer-side legibility from one naturalistic deployment: one specified behavioral constraint, one choreographic work, one internal contrast, one venue, one public audience sample, and one post-performance self-report channel. The analysis estimates associations among reports under that design. The present deployment does not establish the general validity of EtoM across other artistic domains, behavioral systems, or experimental settings. Although BH-FDR correction was applied within the Q10 association family and across the four Q2 sensitivity tests, the broader descriptive and sensitivity analyses should be interpreted as exploratory.

The measurement channel defines the scope of the claim. Post-performance reports support an audience-level profile of recognition, uncertainty, active meaning-making, structural entry, and internal contrast preference. Time-resolved mechanisms of event parsing, attention, prediction, or inference require online measures during exposure. Causal effects of particular construction choices within the EtoM pipeline require controlled variation of those choices across future designs.

Follow-up work can vary the constraint, disclosure condition, contrast placement, continuity-constraint strength, audience, medium, and measurement channel. Online measures such as event-boundary marking, reaction-time probes, gaze, movement, or continuous report could test how observer-side legibility develops during performance. Parallel laboratory and computational implementations could use the same declared-constraint logic in more controlled task-spaces, enabling parameterized tests of how specified organization becomes recoverable under different observation conditions.

A direct future test would compare closely matched movement histories that preserve the operative invariant I(ϕ) with histories in which continuity is deliberately weakened, while holding the observer system and reporting channel constant. Before audience testing, an independent team that did not construct the material would use preregistered criteria to verify that the intended difference was present. Basic surface features—including duration, motion energy, synchrony, sound, and performance quality—would be matched or included in the analysis. Observers would then complete preregistered discrimination and next-event-prediction tasks using new movement histories under concealment, and the analysis would account for variation across both observers and histories. The invariant-preserving condition predicts stronger structural-entry reports, lower uncertainty, stronger active meaning-making, and sharper discrimination between lawful continuation and breakdown. Failure of independent verification would indicate an unsuccessful implementation; successful verification followed by chance-level or reversed observer performance would count against observer-side legibility for that specification and observation channel.

## 5. Conclusion

This field deployment demonstrates a method for studying behavioral constraint legibility under concealment. Across five performances (N = 393), structural-entry reports were associated with lower uncertainty, stronger active meaning-making, and sharper within-work discrimination between constraint-governed material and *Drink Me*, the internal contrast. The deployment remains bounded to one specified constraint, one work, one venue, one audience sample, and one post-performance reporting channel. General validity across other artistic domains, behavioral systems, and experimental settings remains a question for future deployments. Within that scope, the study establishes a tractable design object: a behavioral constraint can govern construction through admissible continuation, remain concealed during observation, and yield a reportable audience-level profile in the realized stream.

## Additional Files

The additional files for this article can be found as follows:

10.5334/joc.515.s1 Supplementary Material S1. Formal Essence-To-Movement (EtoM).

10.5334/joc.515.s2 Supplementary Material S2. SURVEY INSTRUMENT (Q1–Q11; reporting scaffolds under concealment).

10.5334/joc.515.s3 Supplementary Material S3. ESSENCE TRACES (Q11): Trace Banks (Σ(O)) and Equivalence of Form.

10.5334/joc.515.s4 Supplementary Material S4. Zenodo reproducibility package, Version 2.0.0.

## Data Availability

Zenodo: 10.5281/zenodo.18027497 (aggregated results + analysis code). Individual-level questionnaire records and all raw open-ended text responses cannot be shared due to IRB/consent constraints. Aggregated, fully de-identified results and analysis code are available to editors and reviewers via an anonymized repository link provided in the submission system. In accordance with the approved human-subjects protocol and consent language, raw individual-level open-ended responses are not publicly shared.
