# Supplementary material for: Behavioral Constraint Legibility Under Concealment
Source: J Cogn. 2026 Aug 19;9(1):43. doi: 10.5334/joc.515 (PMC13496225; doi:10.5334/joc.515)
Supplement: Supplementary file 1 [file joc-9-1-515-s1.pdf]

SUPPLEMENTARY MATERIAL S1

Formal Essence-To-Movement (EtoM)

Supplementary Material S1 proceeds through three layers. First, the originating pressure is stated from within the medium itself: movement has limited propositional bandwidth, and organization must therefore hold in realized behavior. Second, a cooperative ball-game toy model instantiates the structure at minimal scale, making admissibility, drift, breakdown, concealment, and equivalence of form directly inspectable. Third, Essence-to-Movement (EtoM) is specified as a law-preserving architecture for carrying one governing constraint through formal construction, realized continuation, and observer-side access.

S1.1 Origin and Motivating Pressure

Essence-to-Movement (EtoM) arose in choreographic studio practice in response to a structural communication challenge in dance: overt movement has limited propositional bandwidth. Even when movement adopts relatively "literal" strategies (e.g., conventionalized gesture systems such as classical-ballet pantomime), the medium remains constrained by (i) a finite motor vocabulary, and (ii) the observer's reliance on perceptual organization and event segmentation rather than discrete symbolic composition. Language stabilizes fine-grained propositions—agents, attributes, temporal relations, counterfactuals—via combinatorial reference. Behavior typically cannot do this without external scaffolds (program

notes, keys, explicit rules, narration, or other metadata).

At the opposite pole, non-narrative and highly abstract work increases formal degrees of freedom but reduces propositional specificity further, placing greater demands on an observer's online grouping, prediction, and segmentation capacities. Across the spectrum from referential to abstract, "understanding" is often organized through sensorimotor and perceptual coupling to an evolving behavioral field rather than through lexical decoding. The same constraint appears in domains where organization must be made available through behavior alone: multi-agent simulations, swarm robotics, interactive installations, autonomous control policies, collective animal motion, and human group coordination. In such systems, explicit labels and propositions do not propagate directly through the behavioral medium. What can propagate is structured action under constraint.

EtoM is a framework for constructing and analyzing law-governed behavior under this constraint. A conceptual origin ϕ is treated as a candidate organizing pressure that can be formalized as a behavioral law: a constraint on admissible continuation that must be preserved as conditions change. EtoM specifies how such a law is (i) resolved into an invariant, (ii) decomposed into a finite operation basis, (iii) embedded into rule-governed task-spaces ("vessels"), and (iv) realized as admissible histories over time. Critically, EtoM treats ϕ not as a theme to be illustrated, but as a source of structural obligation that must remain exact while outward forms vary.

S1.2 Illustrative Toy Model: A Cooperative Ball Game

A group of players keeps one shared ball in motion. They throw, catch, adjust spacing, recover from small errors, and continue playing as the arrangement changes. An observer watching from outside can see that some continuations keep the game alive, while drops, arbitrary resets, or unrelated movements break the organization.

Across the sequence, bodies, positions, timing, and ball trajectory change while one rule

governs what can happen next. The model therefore separates surface variation from the organizing condition that determines continuation, drift, and breakdown.

This example gives a compact physical instantiation of EtoM. The originating frame, ϕ, is cooperative play with one shared ball. At this stage, the frame allows several possible organizing constraints: the ball must remain off the ground, each player may hold it for a fixed duration, the same partner may receive a pass after another partner has received one, or all players may keep repositioning while play continues.

For this toy model, the selected Essence is off-ground continuity. The operative invariant, $I(\phi)$, is that the shared ball remains off the ground while play is live. This invariant governs what counts as continuation. A throw, catch, recovery, or repositioning belongs when it keeps off-ground continuity active. A play history in which the ball repeatedly comes to rest on the ground leaves the admissible range.

Abstract Form identifies the minimal functions needed to carry that invariant. In this case, the functions are: generate a catchable throw, receive and stabilize the ball, and reposition so future catches remain possible. These functions are action capacities. They carry off-ground continuity under changing conditions.

Structured Form embeds those functions in task-spaces. One vessel may be a fixed pair exchange at short distance. Another may be a passing circle in which the same partner receives a pass after another partner has received one. A third may be a roaming configuration in which all players move while the ball remains live. Each vessel changes the surface conditions of play, while the same invariant constrains what can happen next.

Embodiment is the realized play history: the actual sequence of throws, catches, misses, recoveries, repositionings, and transitions between vessels. Admissible continuation appears when off-ground continuity remains active across these changes. Drift begins when the players continue moving while off-ground continuity loses control over the next action. Breakdown occurs when the history leaves the admissible range of the construction.

Continuity Layers specify how the game admits a play history into the construction. CL_1

tests the governing law itself: while play is live, the ball remains off the ground. CL_2 tests the source of change: throws, catches, recoveries, spacing adjustments, and role shifts arise from the pressure of keeping play alive. CL_3 tests the active vessel: a pair exchange, passing circle, or roaming configuration keeps its own rules for who may pass, receive, move, and continue. CL_4 tests surface variation: distance, timing, throw arc, recovery speed, and player pathway may change while the same off-ground pattern remains traceable. CL_5 tests the task-space: players, ball, timing, spacing, and local environment remain inside a playable field. Together, the layers determine which play histories belong to $R(\phi)$, the set of realized histories admitted by the construction.

Under concealment, the rule remains construction-side. Observers receive the unfolding play histories through visible action. Observer-side access concerns what they can recover from behavior alone. Detectability appears when they register that the play is organized. Trackability appears when they follow one continuing constraint across different throws, catches, spacing changes, and role shifts. Actionability appears when they anticipate or coordinate with future play. Discriminability appears when they distinguish law-keeping histories from breakdowns, such as repeated drops or arbitrary resets.

Equivalence of form appears when different observers describe the same organization in different words. One observer may say "keep-up," another may say "cooperative juggling," and another may say "never let it drop." The wording differs, while each description tracks the same organizing constraint. Under concealment, that convergence is assessed at the level of form rather than shared vocabulary.

The ball game instantiates the full EtoM construction in a minimal setting. It begins with one originating frame and one selected Essence. It develops an Abstract Form, places that function set into task-spaces, realizes histories in Embodiment, filters those histories through Continuity Layers, preserves construction-side concealment, and supports observer-side access through detectability, trackability, actionability, and discriminability. It also shows how different descriptions can align through equivalence of form.

S1.3 Formal Specification

S1.3.1 Core Specification

Essence-to-Movement (EtoM) is a law-preserving transformation system parameterized by an origin ϕ. For each admissible ϕ, the system-side object is:

$$S(\phi) = (\phi, \mathrm{EtoM}(\phi))$$

where ϕ is a conceptual origin declared for a specific domain of instantiation, and EtoM(ϕ) is the structured transformation sequence through which law-governed behavior is generated from that origin.

EtoM(ϕ) yields Embodiment(ϕ), written R(ϕ):

$$R(\phi) = \{\, r \mid r \text{ is a realized history admitted by } CL_1\text{–}CL_5 \,\}.$$

Observer-side pickup is modeled separately:

$$L(\phi; O) = \mathrm{Legibility}(R(\phi), O)$$

where O denotes an observer system coupled to realized behavior under specified observation conditions. L(ϕ; O) is an observer-relative access relation. Its specification requires that R(ϕ) has already been fixed and that O has been instantiated. L(ϕ; O) is not a component of S(ϕ).

The framework is substrate-independent. The same formal structure applies in choreography, performance analysis, human–computer interaction, multi-agent coordination, simulation, and domain-specific measurement systems, provided that behavior can be organized by internal constraints and presented as histories over time. The provenance of ϕ may lie in empirical data, theoretical constructs, engineered specifications, design briefs, or lived experience. A candidate ϕ supports EtoM only when it can be resolved into a stable behavioral

law and propagated structurally through the full construction.

S1.3.2 Internal Construction

Internally, EtoM(ϕ) proceeds through four linked phases:

$$\text{Essence}(\phi) \to \text{Abstract Form}(\phi) \to \text{Structured Form}(\phi) \to \text{Embodiment}(\phi)$$

The following notation fixes the construction:

**Symbol**	**EtoM phase**	**Formal role**
I(ϕ)	Essence(ϕ)	Behavioral invariant selected or distilled from ϕ
F(ϕ)	Abstract Form(ϕ)	Finite set of law-bearing functions
V(ϕ)	Structured Form(ϕ)	Family of vessels or task-spaces
R(ϕ)	Embodiment(ϕ)	Set of realized histories admitted by CL_1–CL_5

Each phase has a distinct functional role and associated validity conditions. The transformation is law-preserving: no phase may introduce new semantic content.

Each phase refines and propagates the same structural demand encoded in the previous phase. Expressive, symbolic, or thematic material is admissible only when it functions as a pressure-bearing realization of the invariant. Material that functions primarily as representational overlay—signaling what ϕ is "about" without constraining admissible continuation—is excluded by construction.

The construction objects name the stages of EtoM. The construction functions name the processes that carry one stage into the next.

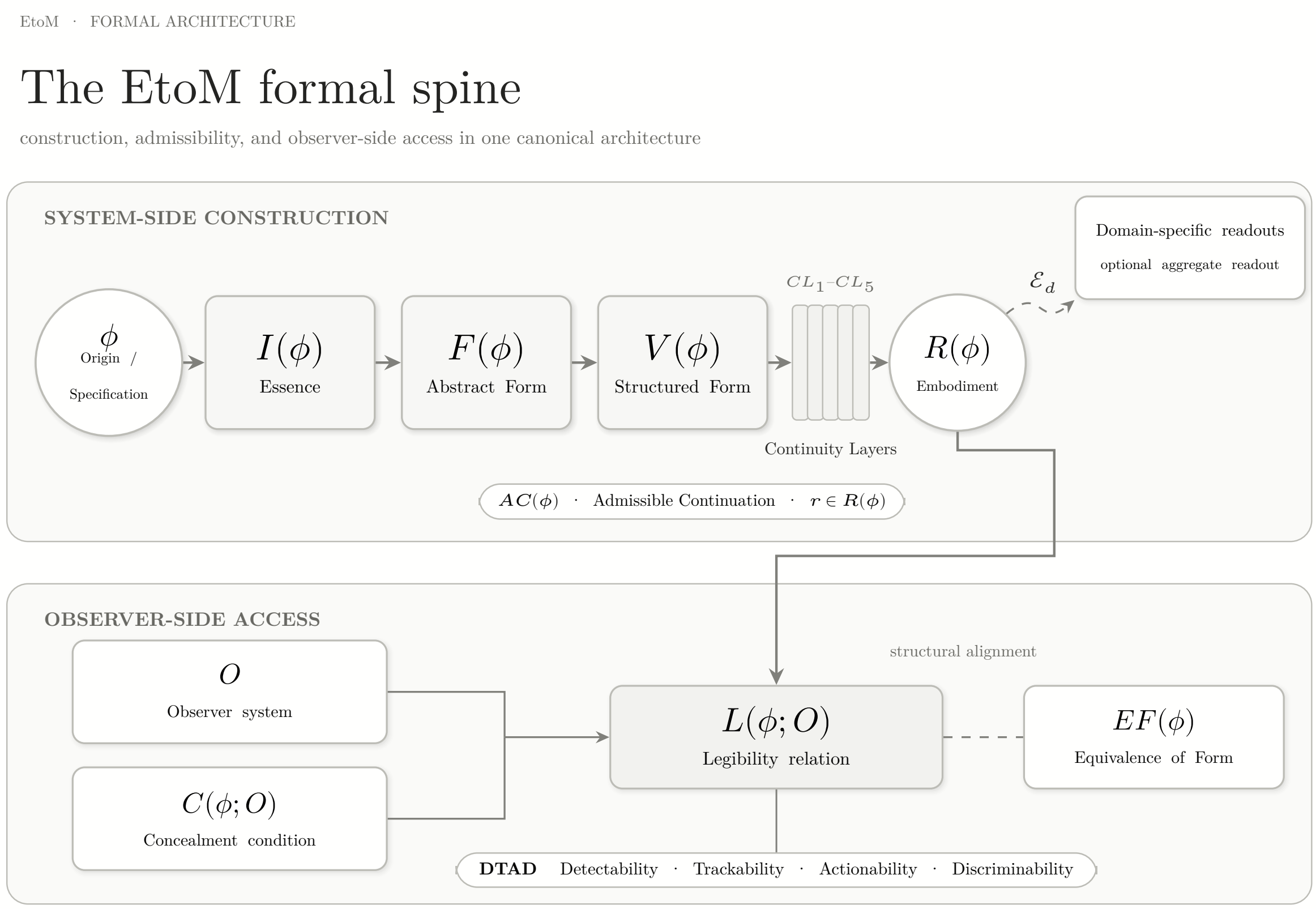

Figure S1.1: Essence-to-Movement architecture: construction sequence and continuity layers.

$$F_1 : \phi \mapsto I(\phi)$$

$$F_2 : I(\phi) \mapsto F(\phi)$$

$$F_3 : (I(\phi), F(\phi)) \mapsto V(\phi)$$

$$F_4 : (I(\phi), F(\phi), V(\phi), \text{CL1–CL5}) \mapsto R(\phi)$$

F_1 selects the operative invariant from the originating frame. F_2 articulates the invariant into the finite operation basis. F_3 embeds the operation basis into rule-governed task-spaces. F_4 admits realized histories through the selected invariant, operation basis, vessels, and Continuity Layers.

The display separates the object sequence from the construction functions. The object sequence is $I(\phi)$, $F(\phi)$, $V(\phi)$, and $R(\phi)$. Within that sequence, $F(\phi)$ denotes Abstract Form. The construction functions F_1–F_4 carry the sequence forward from origin, to invariant, to operation basis, to task-space embedding, and finally to admitted realized histories.

In the display above, $\mapsto$ marks the action of the explicitly named construction functions F_1–F_4. Elsewhere in the chapter, arrows that order a reading sequence remain descriptive until a function is named.

S1.3.3 Admissible Continuation

Admissible continuation, denoted AC(ϕ), is the membership condition produced by EtoM's law-preserving construction. A candidate continuation is admissible when the selected invariant I(ϕ) remains active as timing, spacing, force, role, material, task-space, or local configuration changes. Surface profile may vary sharply; admissibility turns on whether the same governing law still constrains what can follow.

For a realized history r:

r $\in$ R(ϕ) $\Leftrightarrow$ r preserves I(ϕ), uses F(ϕ), unfolds within V(ϕ), and satisfies CL_1–CL_5.

Drift begins when behavior continues while answerability to I(ϕ) slackens, breaks, or yields to another organizing condition.

Breakdown marks failure of admissibility at the level of law-preservation rather than surface difference alone.

Admissible continuation belongs inside the system-side object. I(ϕ), F(ϕ), and V(ϕ) specify the law-bearing conditions of continuation; CL_1–CL_5 filter candidate histories; R(ϕ) contains the histories admitted under those conditions. Observer-side legibility remains downstream as L(ϕ; O), the access relation from realized histories to an observer under specified conditions.

S1.3.4 Phase Validity Conditions

Essence(ϕ): Origin Law / Behavioral Invariant ϕ denotes a domain-level origin, such as a task framing, design intent, or organizing phenomenon. It may initially be heterogeneous or semantically overloaded. Essence(ϕ) is the first internal construct of EtoM: a single behavioral law distilled from ϕ that governs all downstream organization.

Essence(ϕ), denoted I(ϕ), is treated as a structural invariant: a unitary constraint that cannot be partitioned without changing the law.

A candidate I(ϕ) is admissible only when it satisfies all three conditions below.

**Condition**	**Requirement**	**Failure state**
Non-reducibility	I(ϕ) cannot be decomposed into independent sub-laws without loss of coherence.	Removing an essential component changes the law such that identity is no longer preserved.
Stability under ordinary variation	I(ϕ) remains traceable across a declared working range W, including task-space, coordination structure, and allocation of roles or resources.	Change inside W induces a change of law.
Constraint sufficiency	I(ϕ) is sufficient to generate downstream organization in behavioral terms: how the system must continue under pressure.	I(ϕ) depends on narrative tags, stylistic codes, ad hoc cues, labels, or metaphors to preserve the constraint.

Essence(ϕ) is the minimal law under which the system operates. If Essence(ϕ) is inadmissible, ϕ cannot support an EtoM instantiation, since no coherent constraint remains to organize later phases.

A given ϕ may admit more than one admissible invariant I(ϕ). Selecting I(ϕ) is a specification choice made by the responsible researcher, designer, or domain practitioner. EtoM permits multiple admissible specification choices for a given ϕ, provided that the selected I(ϕ) is:

1. stated as an operational constraint;
2. carried forward without semantic expansion;
3. fixed for the instantiation once selected.

The empirical burden then becomes whether that fixed law is preserved in R(ϕ) and becomes legible to observers under the imposed observation conditions.

Abstract Form(ϕ): Functional Decomposition Given an admissible I(ϕ), Abstract Form(ϕ) identifies a finite set of minimal functional capacities that implement the law in a content-independent way:

$$F(\phi) = \{f_1, \ldots, f_n\}$$

Each f_i is a pressure-bearing operation: a way of maintaining, distributing, testing, or carrying I(ϕ) across the working range.

F(ϕ) is admissible only when it satisfies the four conditions below.

Abstract Form(ϕ) defines an operation basis for the invariant. At this stage, the construction moves from unity to plurality: a single law I(ϕ) is re-articulated as finite, irreducible, law-bearing components that collectively preserve the same law. Once F(ϕ) is finalized, the system's degrees of freedom at the function-type level are fixed. Later phases vary configurations, not the basis.

Condition	Requirement	Failure state
Derivation	Each f_i is directly derivable from I(ϕ).	A function introduces new themes, interpretations, or semantic content.
Separability	Each f_i is individually law-relevant. In constrained cases, it can carry a coherent instance of I(ϕ) over limited horizons within W.	A function lacks law-bearing relevance.
Minimal redundancy	Removing any f_i degrades the stability or traceability of I(ϕ) downstream.	A function is superfluous relative to W.
Closure	Once F(ϕ) is fixed, later phases may recombine, schedule, or redistribute functions.	A later phase introduces a new function type.

Structured Form(ϕ): Vessels and Task-Spaces Structured Form(ϕ) embeds F(ϕ) in a family of rule-governed vessels that make the law testable in concrete configurations:

$$V(\phi) = \{V_1, \ldots, V_k\}$$

Each vessel V_j specifies:

1. a task-space, such as spatial layout, resource constraints, interaction topology, or allowable state transitions;
2. internal rules that regulate admissible moves in that space.

V(ϕ) is admissible only when its vessels satisfy the three conditions below.

In concrete instantiations, vessels may be organized into higher-level families, such as symbolic proxies, analogical constraints, conceptual rule systems, progressive schedules, or subtextual constraint patterns. These families are admissible only when they function as constraint-bearing structures, changing what is structurally possible. A candidate vessel that transmits meaning primarily through a conventional key rather than constraint-governed behavior is excluded.

Once F(ϕ) has been embedded into V(ϕ), the structural architecture is fixed. Downstream phases may stress, iterate, and realize the vessels, but altering vessel logic requires

Condition	Requirement	Failure state
Law enforcement	The rules of V_j enforce I(ϕ) as a constraint on admissible behavior.	Behavior violating the law remains admissible.
Cross-vessel compatibility	I(ϕ) remains intact while moving between vessels.	Changing vessels changes the law rather than the conditions.
Structural independence from representational content	Vessels are specified through roles, relations, and constraints rather than narrative content. Different bodies, robots, software modules, or interfaces can inhabit the same structure.	The vessel transmits meaning primarily through narrative content or surface realization.

re-specifying the instantiation.

S1.3.5 Embodiment(ϕ) and Continuity Layers

Embodiment(ϕ) generates concrete realizations: movement sequences, interaction histories, or trajectories. These realizations are subject to a pre-specified stack of Continuity Layers that filters structurally invalid candidates.

Embodiment functions as verification of the prior architecture. Realizations show whether I(ϕ), F(ϕ), and V(ϕ) withstand practical pressures—space, time, noise, and execution variability—while preserving the same law.

Let R(ϕ) denote the set of realized histories admitted by the Continuity Layers. A realization r belongs to R(ϕ) only when it satisfies all five layers.

Together, CL_1–CL_5 define a fixed continuity stack. CL_1 secures a root hold on the law; CL_2 constrains permitted sources of change; CL_3 organizes that change within vessel rules; CL_4 regulates stable surface patterns; CL_5 anchors the realization within an operative environment.

Delays, hesitations, overloads, or near-failures may function as controlled stress tests. They are admissible only when they remain law-governed, reveal how the constraint holds or nearly breaks under pressure, and continue without abandoning the law.

Embodiment(ϕ) is identified with R(ϕ): the set of realized histories satisfying all Conti-

Layer	Acceptance condition	Test
CL_1 Law coherence	The realization keeps a coherent instance of I(ϕ) active.	If the law were removed, the behavior would collapse into unstructured variation relative to W.
CL_2 Endogenous drive	Change arises from law-governed dynamics shaped by vessel constraints.	Arbitrary external tags, narrative shortcuts, or stylistic cues that do not alter admissible continuation are excluded.
CL_3 Structural organization	Components obey the rules of the active vessel V_j and continue to implement F(ϕ).	Agents, roles, resources, or modules remain organized by the vessel rules.
CL_4 Surface pattern stability under variation	Local micro-variation preserves a stable law-consistent pattern.	Independent or contradictory fluctuations that undermine traceability of the invariant are excluded.
CL_5 Task-space coherence	Spatial, temporal, and task-relevant relations remain compatible with I(ϕ) under ordinary perturbations within W.	Perturbations operate within the law rather than breaking it.

nuity Layers for the selected I(ϕ), F(ϕ), and V(ϕ). No new semantic content is introduced at this stage. Expression, symbol, or narrative may appear only as consequences of law-preserving behavior; they are not explanatory grounds for admitting a history into R(ϕ).

S1.3.6 Evaluation of Admitted Histories

Once realized histories have been admitted to $R(\phi)$, a domain may evaluate selected properties of those histories. The evaluation can produce several domain-specific readouts and, where defined, one aggregate readout. Membership in $R(\phi)$ is fixed before these values are produced.

Let d index the domain of instantiation and let $\mathcal{E}_d$ denote its evaluation operator:

$$R(\phi) \xrightarrow{\mathcal{E}_d} (\text{domain-specific readouts}, \text{optional aggregate readout}).$$

An implementation may evaluate one admitted history or a declared grouping drawn

from $R(\phi)$. It fixes what is measured, the unit at which the readouts are reported, and any rule used to form the aggregate.

Domain evaluation provides a system-side measurement of $R(\phi)$ while the four-phase construction and its admission conditions remain fixed. Observer-side legibility remains separate. A system-side readout may be presented through an observation interface; $L(\phi; O)$ characterizes the preserved organization that becomes available to O through that interface.

S1.4 Observer-Side Access

S1.4.1 Concealment and Legibility

Observer-side concepts enter after the system-side construction has fixed R(ϕ).

Given R(ϕ), legibility is defined relative to an observer system O that interacts with or monitors realized behavior:

$$L(\phi; O) = \text{Legibility}(R(\phi), O)$$

When O is fixed by the experimental or applied setting, L(ϕ; O) may be abbreviated as L(ϕ). The dependency chain is:

$$\phi \rightarrow (I(\phi), F(\phi), V(\phi)) \xrightarrow{CL_1\text{–}CL_5} R(\phi) \xrightarrow{O} L(\phi; O)$$

Legibility characterizes which aspects of the preserved invariant I(ϕ) become available to O from histories r $\in$ R(ϕ), under the domain's observation interface and constraints.

Concealment Concealment, denoted C(ϕ; O), specifies which information is available to O. Under concealment, O is coupled only to realized histories r $\in$ R(ϕ) through the relevant observation interface, such as direct perception, interaction logs, or sensor streams. O is not provided with explicit statements of ϕ, I(ϕ), F(ϕ), V(ϕ), or rules functioning as narrative

keys or declarative explanations of what should be tracked.

This observer-side constraint is stricter than label-free or non-disclosed computational entry. A system may operate without step labels, class names, or templates at analytic entry while still instantiating the law internally through I(ϕ), F(ϕ), and V(ϕ). If explicit statements of the governing law or decomposition are supplied to O, observed readout no longer reflects pickup from behavior alone. It becomes confounded by instruction-following, label matching, or compliance with a provided interpretive scaffold.

Dimensions of Legibility At a domain-agnostic level, legibility can be decomposed into structural dimensions.

**Dimension**	**Observer-side question**
Detectability	Does O register that behavior is organized or constrained rather than noise-like or purely stylistic?
Trackability	Can O maintain a stable grip on one coherent tendency across variation and changes of condition?
Actionability	Can O use the observed organization to guide expectations, coordinate actions, or intervene in ways that respect the constraint?
Discriminability	Can O distinguish law-keeping histories within W from histories that violate I(ϕ) or fall outside the intended regime?

Which dimensions are foregrounded is an empirical decision driven by domain constraints and measurement tools. Legibility concerns whether O registers persistence and breakdown of an organizing constraint in observed histories and distinguishes that constraint from incompatible organization.

Together, detectability, trackability, actionability, and discriminability define the DTAD dimension set: the observer-side structural dimensions through which $L(\phi; O)$ is evaluated under the relevant observation condition.

Preservation and Access Structural preservation is system-side. It is an admissibility property of realized histories:

r is admissible only if it preserves one behavioral law across I(ϕ), F(ϕ), V(ϕ), and the CL_1–CL_5 filter defining R(ϕ).

Observer access is interface-side. It is an observer-relative relation grounded in admissible histories:

$$R(\phi) \to L(\phi; O)$$

Preservation is fixed by construction and by the CL_1–CL_5 acceptance stack. Access is the mapping from R(ϕ) to L(ϕ; O), evaluated under stated observation conditions and along the relevant legibility dimensions.

S1.4.2 Equivalence of Form

Equivalence of form, denoted EF(ϕ), is an observer-side equivalence relation indexed to a fixed system-side specification ϕ and its enforced admissibility profile. It names the condition under which different observers, or different interpretive framings, converge at the level of induced relational organization even when surface descriptions or conceptual vocabularies diverge.

Formally, R(ϕ) induces for each observer system O a mapping:

$$\Psi_O : R(\phi) \to \Sigma(O)$$

where $\Sigma(O)$ denotes the space of internal structures, schemata, or organizational templates that O can sustain and apply to incoming behavior.

Two observers O_1 and O_2 may assign different verbal labels or narratives to the same realized history r ∈ R(ϕ), while still matching at the level of the invariant if their induced structures are isomorphic to the same law:

$$I(\phi) \cong \sigma_1 \cong \sigma_2$$

where:

$$\sigma_i = \Psi_{O_i}(r)$$

$$\sigma_i \in \Sigma(O_i)$$

and $\cong$ denotes isomorphism to $I(\phi)$ as realized in $R(\phi)$.

Operationally, equivalence of form is evaluated by structural compatibility with the preserved constraint as enforced in construction. A structure σ is compatible when it tracks law-keeping versus breakdown in a manner consistent with the inclusion–exclusion conditions defining membership in $\mathrm{R}(\phi)$, including the admissibility range implemented by CL_1–CL_5.

What is shared is an organizing constraint, not a word or theme. Observers may register the same pressure across variation while naming it differently. Divergence in labels is compatible with equivalence of form provided that the same invariant is being tracked.

Under concealment, equivalence of form provides an empirical criterion for cross-observer alignment to structure available in $\mathrm{R}(\phi)$. It also marks limits when alignment fails. Descriptive convergence, compression vocabulary, or thematic overlap may be suggestive, but equivalence of form is established only when uptake remains structurally answerable to the same preserved constraint.

The construction, evaluation, and access terms introduced above form one formal architecture. Table S1.1 consolidates the terms governing source specification, admitted realization, optional domain evaluation, observer-side access, and structural uptake.

Table S1.1: EtoM formal taxonomy and access terms

Formal name	Symbol	Position	Function
Origin / Specification	ϕ	Source	Declares the domain object and structural obligation.
Essence	$\mathrm{I}(\phi)$	Law	Fixes the operative invariant.
Abstract Form	$\mathrm{F}(\phi)$	Operation Basis	Articulates the invariant into pressure-bearing capacities.
Structured Form	$\mathrm{V}(\phi)$	Vessel Structure	Embeds the basis in rule-governed task-spaces.
Continuity Layers	CL_1–CL_5	Filter	Tests law-preserving continuation.
Embodiment	$\mathrm{R}(\phi)$	Realization	Contains admitted realized histories.
Admissible Continuation	$\mathrm{AC}(\phi)$	Membership	Marks membership in $\mathrm{R}(\phi)$ under the selected law.
Domain Evaluation	$\mathcal{E}_d$	Post-Admission Evaluation	Optionally produces domain-specific readouts and, where defined, an aggregate readout from admitted histories.
Observer System	O	Observer Side	Identifies the observer system coupled to realized histories under specified observation conditions.
Concealment	$\mathrm{C}(\phi; \mathrm{O})$	Access Condition	Regulates which construction terms are available to O.
Legibility	$\mathrm{L}(\phi; \mathrm{O})$	Observer-Side Relation	Characterizes what preserved organization becomes available to O.
Equivalence of Form	$\mathrm{EF}(\phi)$	Structural Alignment	Marks shared structural uptake across descriptions.
