# Supplementary material for: Behavioral Constraint Legibility Under Concealment
Source: J Cogn. 2026 Aug 19;9(1):43. doi: 10.5334/joc.515 (PMC13496225; doi:10.5334/joc.515)
Supplement: Supplementary file 2 [file joc-9-1-515-s2.pdf]

SUPPLEMENTARY MATERIAL S2

SURVEY INSTRUMENT (Q1–Q11; reporting scaffolds under concealment)

S2.1 Purpose and scope

This supplement provides the verbatim item wording and response formats for the post-performance audience questionnaire (Q1–Q11) administered across five performances (analytic sample N = 393). The instrument is designed for a heterogeneous general-public audience and is optimized for rapid completion (approximately five minutes) on personal devices.

S2.2 Functional role in the study (reporting scaffolds; not law keys)

The questionnaire functions as a reporting scaffold for a movement-only observation channel. It supports non-specialist reporting of organized uptake without requiring dance-analytic vocabulary. Items Q4 (themes/ideas), Q5 (emotions), and Q10 (structural entry routes) provide constrained response options to increase reportability and comparability across respondents, including explicit non-resonance options and an "Other" option where applicable.

S2.3 Concealment and lexical overlap (operational definition)

Concealment is preserved in the instrument design. Respondents are not provided with the system-side law specification (ϕ, $I(\phi)$, $F(\phi)$, $V(\phi)$) or admissibility/continuation rules, and no item supplies a usable instruction set for deriving the law. Incidental lexical overlap between everyday language in the program/instrument and everyday descriptors of experience is expected and is not treated as disclosure. Disclosure is defined operationally by access to system-side specification and constraint-enforcement/admissibility rules, not by the presence of any single word in paratext or response options.

FULL SURVEY INSTRUMENT:

Consent Information for Participation in Research

Title of Study: From Essence to Movement

Researcher: [name redacted for double-blind review]

Institution: [institution redacted for double-blind review]

IRB #: [number redacted for double-blind review]

Thank you for attending our performance and considering participating in this study. This research explores how abstract concepts are communicated through dance.

Participation in this study is entirely voluntary and anonymous. The survey will take approximately 5 minutes to complete, and you may stop at any time. By clicking “Begin,” you consent to participate in this research study.

▢ Begin

Q1. How old are you?

o 11 and younger

o 12–14

o 15–17

o 18–24

o 25–34

o 35–44

o 45–54

o 55 and older

o I prefer not to answer

Q2. What is your experience with dance or performing arts?

o No Experience

o Beginner

o Intermediate

o Advanced

o Professional

Q3. How familiar are you with performances that explore abstract themes and unconventional movement?

o Not familiar at all

o Slightly familiar

o Moderately familiar

o Very familiar

o Extremely familiar

Q4. Which themes or ideas did the performance make you reflect on? *Select all that apply.*

▢ Identity and Transformation

▢ Absurdity and Chaos

▢ Power and Authority

▢ Connection and Isolation

▢ Choices and Consequences

▢ None of these themes reflected my experience

▢ Other (please specify):

Q5. Which emotions did you feel during the performance? *Select all that apply.*

▢ Wonder or Amazement

▢ Tension or Anxiety

▢ Humor

▢ Confusion or Disorientation

▢ Empathy

▢ Discomfort

▢ Did not reflect my experience

▢ Other (please specify):

Q6. How did you engage with the performance? ***Choose the answer that best represents your experience.***

o I focused on its abstract elements rather than a clear story.

o I connected fragmented moments to create my own interpretation.

o My emotional responses shaped how I engaged with the work.

o My intellectual reflections influenced my engagement.

o I experienced it as a cohesive story with a clear progression.

o I'm not sure.

Q7. To what extent did the abstract presentation of the characters (e.g., shifting individuals, groups or voices) reflect aspects of your personal experiences or emotions? ***Choose the answer that best represents your experience.***

o I felt engaged, as if I were actively shaping the meaning of the performance.

o I recognized familiar emotions or experiences in the way the characters were presented.

o I noticed symbolic or abstract connections between the performance and broader ideas.

o I noticed some resonance, but the connection felt distant or minimal.

o I didn't find any connection to the characters or voices presented.

Q8. How much did the performance make you feel like an active participant in creating its meaning? ***Choose the answer that best represents your experience.***

o I felt engaged, as if I were actively shaping the meaning of the performance.

o I engaged actively with many elements.

o I felt somewhat involved in interpreting the themes.

o I noticed moments that invited reflection.

o I observed without feeling involved in meaning-making.

Q9. Which section of the performance resonated with you the most?

o Drink Me (Introduction and the Golden Palace)

o Identity (Fabric and Mutated Transformation)

o Through Cryptic Maze (Living Maze and Shifting Forms)

o Guidance (Red Light/Green Light Game)

o Through Metropolitan City (Individuality and Collectivity)

o Chaos (Tea Party, Soccer Team, and Three Sisters of the Well)

o Dominion (Pas de Quatre and Red/White Roses)

o Authority (The Queen's Dance)

o Undo Me (Finale)

Q10. How did specific elements of the performance resonate with you? *Select all that apply.*

▢ Symbolism: I noticed symbolic elements (e.g., the soccer ball or walker), which carried layered meaning and invited reflection.

▢ Conceptual Layers: I interpreted broader conceptual ideas through scenes (e.g., the shifting maze or the Red Light/Green Light game), which suggested abstract themes for consideration.

▢ Metaphors: I connected to metaphoric representations (e.g., the scarf or the fabric), which embodied ideas through physical analogies.

▢ Subtext: I observed subtle subtext (e.g., the red/white roses painting over mistakes or the tea party), which hinted at hidden layers of meaning beneath the surface.

▢ Progression: I noticed how some parts of the performance started unclear and then made more sense over time.

▢ None of these elements resonated with me.

Q11. What would you identify as the core essence of the performance?

If you were to describe it in one word or phrase, what would it be? Feel free to elaborate.
