# Supplementary material for: Behavioral Constraint Legibility Under Concealment
Source: J Cogn. 2026 Aug 19;9(1):43. doi: 10.5334/joc.515 (PMC13496225; doi:10.5334/joc.515)
Supplement: Supplementary file 3 [file joc-9-1-515-s3.pdf]

SUPPLEMENTARY MATERIAL S3

ESSENCE TRACES (Q11): Trace Banks (Σ(O)) and Equivalence of Form

---

S3.1 Purpose and scope

This supplement presents a curated set of short audience excerpts from the open-ended Essence prompt (Q11), which asked respondents to describe, in their own words, the "core essence" of the performance. Within the EtoM framework, these responses are treated as observer-side language traces $\sigma \in \Sigma(O)$ produced after exposure to the realized performance field $R(\phi)$ under concealment. The trace banks are provided to make concrete the observer-side constructs defined in Supplementary Material S1, especially concealment, legibility, and equivalence of form, by showing the range of surface encodings that arise when the law is not named.

S3.2 Trace definition and curation

A "trace" is a short, non-identifying excerpt distilled from a Q11 response that preserves the respondent's expressed organizing impression and evaluative stance. Traces were prepared by:

(i) removing or abbreviating potentially identifying details;

(ii) lightly normalizing obvious spelling/typographic errors where needed for readability;

(iii) condensing longer responses into compact excerpts while preserving the original meaning and valence.

A single response could yield more than one trace if it contained multiple distinct impressions. Trace IDs index the bank and trace number (e.g., [B4-07]) and do not encode respondent identity, performance night, or submission order.

S3.3 Interpretation under concealment: scope and limits (non-inferential status)

Q11 is optional and collected after the performance; the traces therefore reflect after-the-fact linguistic reconstructions produced in a survey context. They are not treated as direct access to internal mechanisms, and they are not used as evidence that observers shared a single interpretation.

The traces serve three descriptive functions aligned to the Methods section:

1. Illustration of Σ(O): They characterize the diversity of observer-side encodings produced by coupling to realized behavior under concealment.

2. Illustration of equivalence of form: They exemplify how heterogeneous verbal encodings can remain compatible with a preserved organization, while the primary quantitative legibility profile is indexed separately by convergent patterns in Q4–Q10.
3. Boundary marking: Low-contact traces (confusion/overload/rejection) delimit limits on pickup through the observation interface.

S3.4 Trace banks (illustrative selection)

To retain diversity of wording and stance while keeping the supplement readable, each bank reports 15 traces (90 total) selected to span within-bank breadth. Bank membership is descriptive and non-exclusive; ordering is for readability only.

Bank 1 — Constraint and organization: coherence, continuity, tension

Traces here emphasize global organization (often via 'tension', 'order in chaos', or 'held together').

[B1-01] "A Troubled Mind"

[B1-02] "Chaos and Intention can exist at the same time"

[B1-03] "Confrontation"

[B1-04] "Constant chaos"

[B1-05] "Controlled chaos"

[B1-06] "Finding Equilibrium"

[B1-07] "Layered"

[B1-08] "Life is a cacophony"

[B1-09] "Struggling Against Oneself"

[B1-10] "Synergy"

[B1-11] "Systemic Chaos"

[B1-12] "Tension"

[B1-13] "Truth"

[B1-14] "Unity"

[B1-15] "Trying not to break"

Bank 2 — Coupling stance and evaluation: engagement, uptake, rejection

Traces here express the viewer's coupling stance (e.g., captivated, energized, bored, annoyed) and evaluative orientation.

[B2-01] "Abstractly beautiful and powerful"

[B2-02] "Action packed"

[B2-03] "Annoyance. Where was the dancing? It's just bad."

[B2-04] "Craziest ballet I've seen!"

[B2-05] "Electrifying"

[B2-06] "Elaborate"

[B2-07] "Free"

[B2-08] "Intense"

[B2-09] "Nonsense"

[B2-10] "Organized Chaos"

[B2-11] "Powerful"

[B2-12] "Riveting"

[B2-13] "Strength"

[B2-14] "Unique"

[B2-15] "Captivating"

Bank 3 — Affective/phenomenological correlates: felt quality of constraint

Traces here describe the felt phenomenology of the work (e.g., intensity, altered reality, visceral tone) without specifying a narrative.

[B3-01] "Acid"

[B3-02] "Beauty in chaos"

[B3-03] "Beyond basic perception/outside of reality"

[B3-04] "Dissociation"

[B3-05] "Ethereal"

[B3-06] "Feeling of being trapped"

[B3-07] “Finding Beauty in the Chaos of Life”

[B3-08] “Hallucinations”

[B3-09] “Insanity”

[B3-10] “Madness of my mind”

[B3-11] “Psychedelic”

[B3-12] “Raw vulnerability”

[B3-13] “Sensory immersion”

[B3-14] “Dreams and reality”

[B3-15] “Trippy”

Bank 4 — Relational binding and persistence: unity, connection, interdependence

Traces here foreground relations—binding, dependence, togetherness, separation—and persistence of connection through change.

[B4-01] “Cycles!”

[B4-02] “Desire to connect more”

[B4-03] “Emotional Change”

[B4-04] “Emotional differences shaping how the audience feels. In the play there seemed to be a wide variety of emotions between characters which shaped how they each acted.”

[B4-05] “Escapism”

[B4-06] “Inside”

[B4-07] “Introspection”

[B4-08] “New Experience”

[B4-09] “Opportunity to engage with my own transformations. My husband passed almost 6 yrs ago… I have continued to think about it since my viewing.”

[B4-10] “Seeking”

[B4-11] “Self-searching”

[B4-12] “Spiraling”

[B4-13] “Transformation through movement”

[B4-14] “Wonder”

[B4-15] “Togetherness”

Bank 5 — Overload and breakdown markers: near-failure, loss of contact

Traces here report overload, disorientation, or breakdown of sense-making; they function as negative cases for legibility.

[B5-01] “Chaotic, everything happening all at once”

[B5-02] “Disillusion: that the brain that is in overdrive becomes disillusioned”

[B5-03] “Dystopian”

[B5-04] “Heart attack”

[B5-05] “Hitting rock bottom”

[B5-06] “I have no idea I was confused the entire time.”

[B5-07] “Mayhem”

[B5-08] “Mish Mash”

[B5-09] “Perplexing”

[B5-10] “Somuchwork”

[B5-11] “The Octopus, a birth?”

[B5-12] “Uhhhhm”

[B5-13] “Unpredictable”

[B5-14] “Too much!”

[B5-15] “Overwhelming”

Bank 6 — Epistemic openness and questioning: meaning-making stance

Traces here frame the experience as inquiry: searching, questioning, noticing hidden layers, or reflecting on interpretation itself.

[B6-01] “Decisions - so many to make, which one is the right one, is there a right one”

[B6-02] “Mind-bending”

[B6-03] “More than meets the eye”

[B6-04] “Open ended”

[B6-05] “Power dynamics and how they can be illogical or arbitrary”

[B6-06] “Questioning”

[B6-07] “Subversion”

[B6-08] “The unconscious”

[B6-09] “Uncertainty in times of change”

[B6-10] “Understanding abstract movements to gain a deeper meaning”

[B6-11] “Unknowingness”

[B6-12] “Up for interpretation”

[B6-13] “Exploratory”

[B6-14] “Interpretive”

[B6-15] “Reflection”
