# Supplementary material for: Behavioral Constraint Legibility Under Concealment
Source: J Cogn. 2026 Aug 19;9(1):43. doi: 10.5334/joc.515 (PMC13496225; doi:10.5334/joc.515)
Supplement: Supplementary file 4 [file joc-9-1-515-s4.pdf]

aggregated_results_release

Zenodo reproducibility package, Version 2.0.0

codebook.csv

```
variable,description,type,levels_or_coding
Q1_age_group,Age group,categorical,11 and younger; 12–14; 15–17; 18–24; 25–34; 35–44; 45–54; 55 and older; I prefer not to answer
Q1_collapsed,Collapsed age sensitivity grouping,categorical,Under 25 = Q1 categories through 18–24; 25 and older = Q1 categories 25–34 through 55 and older; undisclosed age excluded
Q2_dance_experience,Dance/performing-arts experience,categorical,No Experience; Beginner; Intermediate; Advanced; Professional
Q3_familiarity_abstraction,Familiarity with abstract/unconventional performance,categorical,Not familiar at all; Slightly familiar; Moderately familiar; Very familiar; Extremely familiar
Q4_recognized,Thematic recognition,binary,"1 = any substantive option, including Other; 0 = explicit non-recognition as sole endorsement"
Q5_recognized,Affective recognition,binary,"1 = any substantive option, including Other; 0 = explicit non-recognition as sole endorsement"
Q6_uncertain,Uncertainty endpoint,binary,"1 = ""I’m not sure.""; 0 = Q6 responses 1–5"
Q7_resonance_any,Personal resonance present,binary,1 = Q7 responses 1–4; 0 = no-connection endpoint
Q8_active_any,Active meaning-making present,binary,1 = Q8 responses 1–4; 0 = observed-only endpoint
Q9_drink_me,Internal-contrast preference,binary,1 = Drink Me; 0 = any EtoM-governed section
Q10_structural_entry,Structural-entry report,binary,1 = at least one listed structural descriptor endorsed; 0 = only explicit non-resonance option
RR_Q10,Risk ratio for Q10 association,continuous,Outcome risk in Q10 Yes divided by outcome risk in Q10 No; 0.5 added to all four cells only when a zero cell occurs
BH_Q10,BH-FDR adjusted value across six Q10 tests,continuous,Benjamini–Hochberg correction across Q10 × Q4–Q9
FFH_Q2,Q2 sensitivity exact test,continuous,Two-sided Fisher–Freeman–Halton exact test for each 5×2 table
BH_Q2,BH-FDR adjusted value across four Q2 tests,continuous,Benjamini–Hochberg correction across Q2 × Q10/Q6/Q8/Q9
```

LICENSE.txt

LICENSE

Aggregated outputs and documentation
Creative Commons Attribution 4.0 International (CC BY 4.0)

You may share and adapt the aggregated outputs and documentation for any purpose, provided appropriate credit is given and changes are indicated.

Source code
MIT License

Copyright (c) 2026 The Authors

Permission is hereby granted, free of charge, to any person obtaining a copy of this software and associated documentation files, to deal in the Software without restriction, including without limitation the rights to use, copy, modify, merge, publish, distribute, sublicense, and/or sell copies, subject to inclusion of this copyright notice and permission notice.

THE SOFTWARE IS PROVIDED "AS IS", WITHOUT WARRANTY OF ANY KIND, EXPRESS OR IMPLIED, INCLUDING MERCHANTABILITY, FITNESS FOR A PARTICULAR PURPOSE, AND NONINFRINGEMENT. IN NO EVENT SHALL THE AUTHORS OR COPYRIGHT HOLDERS BE LIABLE FOR ANY CLAIM, DAMAGES, OR OTHER LIABILITY ARISING FROM, OUT OF, OR IN CONNECTION WITH THE SOFTWARE OR ITS USE.

manifest.csv

```
file,size_bytes,sha256
LICENSE.txt,1016,8a13d098eaae11709b4a16599980322f3ea231cea5cd2e5b6d599ffc59aaac94
README.txt,2580,0020f23d3b0c8575eaf197afc4c2a59e145b6fc803070e4cd8e9c4d67df506ac
codebook.csv,2005,8fba8904ed70d434dfd574a3fac7d00e7eb03ab16c1b80b21b5199d273f9a9ee
missingness_by_item.csv,173,cd8a6baa42e0516786d31f3596af79a20720d3de07d9f2fb6571c6ee00647394
outputs/collapsed_age_fisher.csv,132,d412bb8b02b9e0d911ec91c2bde7dc9810bbe91d5949e8cdec0c7bdbc1ad266d
outputs/q10_q4_q9_associations.csv,1196,0110b5b456146291c41cb44cf712defccbff8559724c0b2bd5ab504352f04520
outputs/q1_q3_by_q6_crosstabs.csv,787,4d5cd8b161faea6c135c93395144101fb1f30c2053aa8bcf5c6e77b2380165dd
outputs/q1_q3_by_q6_tests.csv,357,19c6d39218e096ae693340a7da05c41656bcd2dd7039acb7c2b56e802c442b62
outputs/q2_sensitivity_ffh.csv,1090,c0ce5eccdbc83b25896da8638f315bcb7d428ea1333fca8e41d5ef9fb7517f15
outputs/risk_ratios_and_95CIs.csv,613,561f44b0dab0b95769aebd267ecf6429102573d8a6629a229231b324242c2009
outputs/summary_counts.csv,470,50d7ceb046733f231c01215274daa2b2c1e5d6a0532b53b4dbd505edad298b7e
outputs/summary_proportions.csv,1191,839ce66c52cb31b70b04c114dbfec965e283cb8ec3d9b6a18d532adf6e7ab54d
outputs/table10.csv,1090,c0ce5eccdbc83b25896da8638f315bcb7d428ea1333fca8e41d5ef9fb7517f15
outputs/table9.csv,1196,0110b5b456146291c41cb44cf712defccbff8559724c0b2bd5ab504352f04520
outputs/tests.csv,1315,91edae74ecb747629ec141015f858e6a948009b2feafcb609c5f36c4dead252e
qa_alignment_check.csv,3054,d079a9a4a8af5927bb30ba98e9fefb3d6308ce753e540651feefbd87537dae44
scripts/make_aggregates.py,18988,3ecf89a1d1bf0bac62ca7a78278f2e74cdb6b3b4296b5ea0341bdf6d6895b27d
scripts/requirements.txt,66,7efb75158cf4bff30ade3dedce97f8cba1694a47946eb290f26247f12a335559
```

missingness_by_item.csv

```
item,answered_n,missing_n,missing_percent
Q1,393,0,0.0
Q2,393,0,0.0
Q3,393,0,0.0
Q4,393,0,0.0
Q5,393,0,0.0
Q6,393,0,0.0
Q7,393,0,0.0
Q8,393,0,0.0
Q9,393,0,0.0
Q10,393,0,0.0
```

outputs/collapsed_age_fisher.csv

```
age_group,engaged,uncertain,total,fisher_two_sided_p
Under 25,284,23,307,0.8176067429136975
25 and older,77,7,84,0.8176067429136975
```

outputs/q10_q4_q9_associations.csv

```
outcome,q10_no_n,q10_no_N,q10_no_percent,q10_yes_n,q10_yes_N,q10_yes_percent,risk_ratio_yes_vs_no,rr_95ci_low,rr_95ci_high,rr_uses_0.5_correction,fisher_two_sided_p,bh_fdr_q_across_6
Q4: Thematic recognition,11,32,34.375,361,361,100.0,2.865601729521979,1.7972399315605458,4.569046752210291,True,3.7409498040877824e-27,2.2445698824526694e-26
Q5: Affective recognition,29,32,90.625,358,361,99.16897506925208,1.094278345591747,0.9784920248791594,1.223765822494968,False,0.008321315203370079,0.008321315203370079
Q6: Uncertainty,8,32,25.0,23,361,6.371191135734072,0.2548476454293629,0.12421025479596069,0.5228821282717661,False,0.0016638155086252356,0.0019965786103502826
Q7: Personal resonance (any),20,32,62.5,330,361,91.41274238227147,1.4626038781163435,1.1162594120693705,1.9164094664296774,False,2.948620040527483e-05,4.4229300607912246e-05
Q8: Active meaning-making (any),12,32,37.5,303,361,83.93351800554017,2.2382271468144044,1.4277708970837588,3.50872872599466,False,3.282597906820172e-08,6.565195813640344e-08
"Q9: Preference = ""Drink Me""",13,32,40.625,8,361,2.21606648199446,0.05454932878755593,0.024434380959214713,0.12178042390923373,False,6.873119232675497e-11,2.0619357698026492e-10
```

outputs/q1_q3_by_q6_crosstabs.csv

```
variable,category,engaged,uncertain,total,engagement_rate
Q1,11 and younger,1,0,1,1.0
Q1,12-14,2,0,2,1.0
Q1,15-17,8,3,11,0.7272727272727273
Q1,18-24,273,20,293,0.931740614334471
Q1,25-34,9,0,9,1.0
Q1,35-44,8,1,9,0.8888888888888888
Q1,45-54,25,3,28,0.8928571428571429
Q1,55 and older,35,3,38,0.9210526315789473
Q1,I prefer not to answer,1,1,2,0.5
Q2,No Experience,91,9,100,0.91
Q2,Beginner,118,12,130,0.9076923076923077
Q2,Intermediate,89,1,90,0.9888888888888889
Q2,Advanced,45,9,54,0.8333333333333334
Q2,Professional,19,0,19,1.0
Q3,Not familiar at all,116,14,130,0.8923076923076924
Q3,Slightly familiar,132,6,138,0.9565217391304348
Q3,Moderately familiar,67,5,72,0.9305555555555556
Q3,Very familiar,36,5,41,0.8780487804878049
Q3,Extremely familiar,11,1,12,0.9166666666666666
```

outputs/q1_q3_by_q6_tests.csv

```
test,test_type,statistic,df,p_value,effect_size,effect_label
Q1 × Q6,Pearson chi-square,12.4894037171476,8,0.130667099409169,0.1782684863465614,Cramér's V
Q2 × Q6,Pearson chi-square,13.536112923789807,4,0.00893270103405652,0.1855883493905008,Cramér's V
Q3 × Q6,Pearson chi-square,5.003983890681269,4,0.28688896732868174,0.11283963593014652,Cramér's V
```

outputs/q2_sensitivity_ffh.csv

```
outcome,no_experience_n,no_experience_N,no_experience_percent,beginner_n,beginner_N,beginner_percent,intermediate_n,intermediate_N
,intermediate_percent,advanced_n,advanced_N,advanced_percent,professional_n,professional_N,professional_percent,fisher_freeman_hal
ton_two_sided_p,bh_fdr_q_across_4,interpretation
Q10 structural-entry
reports,94,100,94.0,115,130,88.46153846153847,83,90,92.22222222222223,51,54,94.44444444444444,18,19,94.73684210526316,0.5840764294
68547,0.7787685726247293,No simple expertise gradient
Q6
uncertainty,9,100,9.0,12,130,9.23076923076923,1,90,1.1111111111111112,9,54,16.666666666666668,0,19,0.0,0.005215417055108691,0.0208
61668220434764,"Mixed, non-monotonic pattern"
Q8 active meaning-
making,73,100,73.0,102,130,78.46153846153847,79,90,87.77777777777777,44,54,81.48148148148148,17,19,89.47368421052632,0.10363972279
72843,0.2072794455945686,Not significant after BH-FDR
Q9 Drink Me
preference,5,100,5.0,7,130,5.384615384615385,6,90,6.666666666666667,2,54,3.7037037037037037,1,19,5.2631578947368425,0.969709309926
4331,0.9697093099264331,No simple expertise gradient
```

outputs/risk_ratios_and_95CIs.csv

```
outcome,risk_ratio_yes_vs_no,rr_95ci_low,rr_95ci_high,rr_uses_0.5_correction
Q4: Thematic recognition,2.865601729521979,1.7972399315605458,4.569046752210291,True
Q5: Affective recognition,1.094278345591747,0.9784920248791594,1.223765822494968,False
Q6: Uncertainty,0.2548476454293629,0.12421025479596069,0.5228821282717661,False
Q7: Personal resonance (any),1.4626038781163435,1.1162594120693705,1.9164094664296774,False
Q8: Active meaning-making (any),2.2382271468144044,1.4277708970837588,3.50872872599466,False
"Q9: Preference = ""Drink Me""",0.05454932878755593,0.024434380959214713,0.12178042390923373,False
```

outputs/summary_counts.csv

```
measure,count,denominator
Q6 Engaged (responses 1–5),362,393
"Q6 Uncertain (""I’m not sure"")",31,393
Q4 Thematic recognition,372,393
Q5 Affective recognition,387,393
Q7 No connection endpoint,43,393
Q8 Observed-only endpoint,78,393
Q9 Drink Me preference,21,393
Q10 Structural-entry Yes,361,393
Q10 Structural-entry No,32,393
Table 8 recognition-domain endpoint profile,0,393
Table 8 engagement-domain endpoint profile,9,393
Table 8 complete endpoint profile,1,393
```

outputs/summary_proportions.csv

```
measure,count,denominator,proportion,wilson_95ci_low,wilson_95ci_high
Q6 Engaged (responses 1–5),362,393,0.9211195928753181,0.8902111238435584,0.9438751154915224
"Q6 Uncertain (""I’m not sure"")",31,393,0.07888040712468193,0.05612488450847771,0.10978887615644173
Q4 Thematic recognition,372,393,0.9465648854961832,0.9196965183203345,0.9647876810954157
Q5 Affective recognition,387,393,0.9847328244274809,0.9670965169387375,0.9929846225980168
Q7 No connection endpoint,43,393,0.10941475826972011,0.08225129838881196,0.14414001437291094
Q8 Observed-only endpoint,78,393,0.1984732824427481,0.16204182467100206,0.2407423483730641
Q9 Drink Me preference,21,393,0.05343511450381679,0.03521231890458437,0.08030348167966554
Q10 Structural-entry Yes,361,393,0.9185750636132316,0.887312221589277,0.9417342217377367
Q10 Structural-entry No,32,393,0.08142493638676845,0.058265778262263423,0.112687778410723
Table 8 recognition-domain endpoint profile,0,393,0.0,0.0,0.009680084414843918
Table 8 engagement-domain endpoint profile,9,393,0.022900763358778626,0.012094017818359,0.042944230669087494
Table 8 complete endpoint profile,1,393,0.002544529262086514,0.0004493131988420277,0.014270567224068843
```

outputs/table10.csv

```
outcome,no_experience_n,no_experience_N,no_experience_percent,beginner_n,beginner_N,beginner_percent,intermediate_n,intermediate_N
,intermediate_percent,advanced_n,advanced_N,advanced_percent,professional_n,professional_N,professional_percent,fisher_freeman_hal
ton_two_sided_p,bh_fdr_q_across_4,interpretation
Q10 structural-entry
reports,94,100,94.0,115,130,88.46153846153847,83,90,92.22222222222223,51,54,94.44444444444444,18,19,94.73684210526316,0.5840764294
68547,0.7787685726247293,No simple expertise gradient
Q6
uncertainty,9,100,9.0,12,130,9.23076923076923,1,90,1.1111111111111112,9,54,16.666666666666668,0,19,0.0,0.005215417055108691,0.0208
61668220434764,"Mixed, non-monotonic pattern"
Q8 active meaning-
making,73,100,73.0,102,130,78.46153846153847,79,90,87.77777777777777,44,54,81.48148148148148,17,19,89.47368421052632,0.10363972279
72843,0.2072794455945686,Not significant after BH-FDR
Q9 Drink Me
preference,5,100,5.0,7,130,5.384615384615385,6,90,6.666666666666667,2,54,3.7037037037037037,1,19,5.2631578947368425,0.969709309926
4331,0.9697093099264331,No simple expertise gradient
```

outputs/table9.csv

```
outcome,q10_no_n,q10_no_N,q10_no_percent,q10_yes_n,q10_yes_N,q10_yes_percent,risk_ratio_yes_vs_no,rr_95ci_low,rr_95ci_high,rr_uses
_0.5_correction,fisher_two_sided_p,bh_fdr_q_across_6
Q4: Thematic
recognition,11,32,34.375,361,361,100.0,2.865601729521979,1.7972399315605458,4.569046752210291,True,3.7409498040877824e-
27,2.2445698824526694e-26
Q5: Affective
recognition,29,32,90.625,358,361,99.16897506925208,1.094278345591747,0.9784920248791594,1.223765822494968,False,0.0083213152033700
79,0.008321315203370079
Q6:
Uncertainty,8,32,25.0,23,361,6.371191135734072,0.2548476454293629,0.12421025479596069,0.5228821282717661,False,0.00166381550862523
56,0.0019965786103502826
Q7: Personal resonance
(any),20,32,62.5,330,361,91.41274238227147,1.4626038781163435,1.1162594120693705,1.9164094664296774,False,2.948620040527483e-
05,4.4229300607912246e-05
Q8: Active meaning-making
(any),12,32,37.5,303,361,83.93351800554017,2.2382271468144044,1.4277708970837588,3.50872872599466,False,3.282597906820172e-
08,6.565195813640344e-08
"Q9: Preference = ""Drink
Me""",13,32,40.625,8,361,2.21606648199446,0.05454932878755593,0.024434380959214713,0.12178042390923373,False,6.873119232675497e-
11,2.0619357698026492e-10
```

outputs/tests.csv

```
analysis_family,test,test_type,p_value,bh_fdr_q
Age sensitivity,Collapsed under-25 versus 25-and-older × Q6,Fisher exact (two-sided),0.8176067429136975,
Q10 associations,Q4: Thematic recognition,Fisher exact (two-sided),3.7409498040877824e-27,2.2445698824526694e-26
Q10 associations,Q5: Affective recognition,Fisher exact (two-sided),0.008321315203370079,0.008321315203370079
Q10 associations,Q6: Uncertainty,Fisher exact (two-sided),0.0016638155086252356,0.0019965786103502826
Q10 associations,Q7: Personal resonance (any),Fisher exact (two-sided),2.948620040527483e-05,4.4229300607912246e-05
Q10 associations,Q8: Active meaning-making (any),Fisher exact (two-sided),3.282597906820172e-08,6.565195813640344e-08
Q10 associations,"Q9: Preference = ""Drink Me""",Fisher exact (two-sided),6.873119232675497e-11,2.0619357698026492e-10
Q2 sensitivity,Q10 structural-entry reports,Fisher–Freeman–Halton exact (two-sided),0.584076429468547,0.7787685726247293
Q2 sensitivity,Q6 uncertainty,Fisher–Freeman–Halton exact (two-sided),0.005215417055108691,0.020861668220434764
Q2 sensitivity,Q8 active meaning-making,Fisher–Freeman–Halton exact (two-sided),0.1036397227972843,0.2072794455945686
Q2 sensitivity,Q9 Drink Me preference,Fisher–Freeman–Halton exact (two-sided),0.9697093099264331,0.9697093099264331
```

qa_alignment_check.csv

```
check,observed,expected,tolerance,result
Analytic N,393.0,393.0,0.0,PASS
Table 2 Q6 engaged count,362.0,362.0,0.0,PASS
Table 2 Q6 uncertain count,31.0,31.0,0.0,PASS
Table 6 Q4 recognition count,372.0,372.0,0.0,PASS
Table 6 Q5 recognition count,387.0,387.0,0.0,PASS
Table 7 Q7 no-connection count,43.0,43.0,0.0,PASS
Table 7 Q8 observed-only count,78.0,78.0,0.0,PASS
Table 8 recognition-domain endpoint count,0.0,0.0,0.0,PASS
Table 8 engagement-domain endpoint count,9.0,9.0,0.0,PASS
Table 8 complete endpoint count,1.0,1.0,0.0,PASS
Collapsed age Fisher p,0.8176067429136975,0.8176067429,5e-11,PASS
Table 9 row 1 Q10 No numerator,11.0,11.0,0.0,PASS
Table 9 row 1 Q10 Yes numerator,361.0,361.0,0.0,PASS
Table 9 row 1 RR rounded to 2 decimals,2.87,2.87,0.0,PASS
Table 9 row 1 Fisher exact p,3.7409498040877824e-27,3.7409498040877824e-27,1e-14,PASS
Table 9 row 1 BH-FDR q,2.2445698824526694e-26,2.2445698824526694e-26,1e-14,PASS
Table 9 row 2 Q10 No numerator,29.0,29.0,0.0,PASS
Table 9 row 2 Q10 Yes numerator,358.0,358.0,0.0,PASS
Table 9 row 2 RR rounded to 2 decimals,1.09,1.09,0.0,PASS
Table 9 row 2 Fisher exact p,0.008321315203370079,0.008321315203370079,1e-14,PASS
Table 9 row 2 BH-FDR q,0.008321315203370079,0.008321315203370079,1e-14,PASS
Table 9 row 3 Q10 No numerator,8.0,8.0,0.0,PASS
Table 9 row 3 Q10 Yes numerator,23.0,23.0,0.0,PASS
Table 9 row 3 RR rounded to 2 decimals,0.25,0.25,0.0,PASS
Table 9 row 3 Fisher exact p,0.0016638155086252356,0.0016638155086252356,1e-14,PASS
Table 9 row 3 BH-FDR q,0.0019965786103502826,0.0019965786103502826,1e-14,PASS
Table 9 row 4 Q10 No numerator,20.0,20.0,0.0,PASS
Table 9 row 4 Q10 Yes numerator,330.0,330.0,0.0,PASS
Table 9 row 4 RR rounded to 2 decimals,1.46,1.46,0.0,PASS
Table 9 row 4 Fisher exact p,2.948620040527483e-05,2.948620040527483e-05,1e-14,PASS
Table 9 row 4 BH-FDR q,4.4229300607912246e-05,4.4229300607912246e-05,1e-14,PASS
Table 9 row 5 Q10 No numerator,12.0,12.0,0.0,PASS
Table 9 row 5 Q10 Yes numerator,303.0,303.0,0.0,PASS
Table 9 row 5 RR rounded to 2 decimals,2.24,2.24,0.0,PASS
Table 9 row 5 Fisher exact p,3.282597906820172e-08,3.282597906820172e-08,1e-14,PASS
Table 9 row 5 BH-FDR q,6.565195813640344e-08,6.565195813640344e-08,1e-14,PASS
Table 9 row 6 Q10 No numerator,13.0,13.0,0.0,PASS
Table 9 row 6 Q10 Yes numerator,8.0,8.0,0.0,PASS
Table 9 row 6 RR rounded to 2 decimals,0.05,0.05,0.0,PASS
Table 9 row 6 Fisher exact p,6.873119232675497e-11,6.873119232675497e-11,1e-14,PASS
Table 9 row 6 BH-FDR q,2.0619357698026492e-10,2.061935769802649e-10,1e-14,PASS
Table 10 row 1 FFH p manuscript rounding,0.5841,0.5841,0.0,PASS
Table 10 row 1 BH-FDR q manuscript rounding,0.7788,0.7788,0.0,PASS
Table 10 row 2 FFH p manuscript rounding,0.0052,0.0052,0.0,PASS
Table 10 row 2 BH-FDR q manuscript rounding,0.0209,0.0209,0.0,PASS
Table 10 row 3 FFH p manuscript rounding,0.1036,0.1036,0.0,PASS
Table 10 row 3 BH-FDR q manuscript rounding,0.2073,0.2073,0.0,PASS
Table 10 row 4 FFH p manuscript rounding,0.9697,0.9697,0.0,PASS
Table 10 row 4 BH-FDR q manuscript rounding,0.9697,0.9697,0.0,PASS
```

README.txt

README

Behavioral Constraint Legibility Under Concealment
Final aggregated reproducibility package

Purpose
This release provides aggregated outputs, documentation, and analysis code supporting the final Results section of the manuscript. It permits verification of reported counts, proportions, confidence intervals, exact tests, risk ratios, multiple-comparison corrections, and Tables 9 and 10 without distributing row-level response records.

Input
The analysis script takes the authoritative analytic dataset used for the final manuscript analyses as a row-level CSV supplied through the --input argument. The input dataset is not included in this public package.

Analytic sample
N = 393 retained respondents. Q1–Q10 are complete for all retained respondents. Q11 is open-ended, descriptive, and outside this quantitative release.

Analyses reproduced
1. Q6 engagement and uncertainty counts, percentages, and Wilson 95% confidence intervals.
2. Q1–Q3 × Q6 descriptive crosstabs and Pearson chi-square sensitivity summaries.
3. Collapsed under-25 versus 25-and-older two-sided Fisher exact test, excluding the undisclosed-age category.
4. Six Q10 × Q4–Q9 two-sided Fisher exact tests.
5. Risk ratios and log-scale 95% confidence intervals for the six Q10 associations.
6. A 0.5 correction applied to all four 2×2 cells for RR/CI calculation only when a zero cell occurs.
7. Benjamini–Hochberg FDR correction across the six Q10 tests.
8. Four Q2 × outcome Fisher–Freeman–Halton exact tests for Q10, Q6, Q8, and Q9.
9. Benjamini–Hochberg FDR correction across the four Q2 tests.
10. Current Table 9 and Table 10 outputs.
11. Numeric QA checks against the final manuscript values and table numbering.

Run
From the directory containing the authoritative analytic CSV:

```
python scripts/make_aggregates.py --input /path/to/analytic_data.csv --outdir /path/to/output_release
```

Dependencies
Install the versions specified in scripts/requirements.txt.

Contents
README.txt
LICENSE.txt
codebook.csv
missingness_by_item.csv
qa_alignment_check.csv
scripts/make_aggregates.py
scripts/requirements.txt
outputs/summary_counts.csv
outputs/summary_proportions.csv
outputs/q1_q3_by_q6_crosstabs.csv
outputs/q1_q3_by_q6_tests.csv
outputs/collapsed_age_fisher.csv
outputs/q10_q4_q9_associations.csv
outputs/risk_ratios_and_95CIs.csv
outputs/q2_sensitivity_ffh.csv
outputs/table9.csv
outputs/table10.csv
outputs/tests.csv

Data boundary
The package contains aggregated results only. It contains no row-level data, response identifiers, timestamps, or open-ended Q11 responses.

scripts/make_aggregates.py

```
#!/usr/bin/env python3
"""
Generate the public aggregated reproducibility package for:
Behavioral Constraint Legibility Under Concealment

The input is the authoritative analytic dataset used for the final manuscript
analyses. The row-level input is not copied into the output directory.
"""

from __future__ import annotations

import argparse
import csv
import math
from pathlib import Path
from typing import Callable, Iterable

import numpy as np
import pandas as pd
from scipy import stats
from statsmodels.stats.multitest import multipletests


Q1_LEVELS = [
    "11 and younger", "12–14", "15–17", "18–24",
    "25–34", "35–44", "45–54", "55 and older",
    "I prefer not to answer",
]
Q2_LEVELS = ["No Experience", "Beginner", "Intermediate", "Advanced", "Professional"]
Q3_LEVELS = [
    "Not familiar at all", "Slightly familiar", "Moderately familiar",
    "Very familiar", "Extremely familiar",
]

UNCERTAIN = "I’m not sure."
Q4_NONREC = "None of these themes reflected my experience"
Q5_NONREC = "Did not reflect my experience"
Q7_NO = "I didn’t find any connection to the characters or voices presented."
Q8_OBS = "I observed without feeling involved in meaning-making."
Q10_NO = "None of these elements resonated with me."


def wilson_ci(k: int, n: int, alpha: float = 0.05) -> tuple[float, float]:
    if n == 0:
        return float("nan"), float("nan")
    z = stats.norm.ppf(1 - alpha / 2)
    p = k / n
    den = 1 + z * z / n
    center = (p + z * z / (2 * n)) / den
    half = z * math.sqrt((p * (1 - p) + z * z / (4 * n)) / n) / den
    return center - half, center + half


def rr_log_ci(a_no: int, b_no: int, c_yes: int, d_yes: int) -> tuple[float, float, float, bool]:
    a, b, c, d = map(float, (a_no, b_no, c_yes, d_yes))
    corrected = min(a, b, c, d) == 0
    if corrected:
        a, b, c, d = a + 0.5, b + 0.5, c + 0.5, d + 0.5
    rr = (c / (c + d)) / (a / (a + b))
    se = math.sqrt((1 / c) - (1 / (c + d)) + (1 / a) - (1 / (a + b)))
    z = stats.norm.ppf(0.975)
    return rr, math.exp(math.log(rr) - z * se), math.exp(math.log(rr) + z * se), corrected


def chi2_cramers_v(table: np.ndarray) -> tuple[float, int, float, float]:
    chi2, p, dof, _ = stats.chi2_contingency(table, correction=False)
    n = table.sum()
    r, c = table.shape
    v = math.sqrt(chi2 / (n * min(r - 1, c - 1)))
    return chi2, dof, p, v


def logcomb(n: int, k: int) -> float:
    if k < 0 or k > n:
        return float("-inf")
    return math.lgamma(n + 1) - math.lgamma(k + 1) - math.lgamma(n - k + 1)


def fisher_freeman_halton_exact(row_totals: list[int], successes: list[int], tol: float = 1e-12) -> float:
    """Two-sided Fisher-Freeman-Halton exact p-value for an R×2 table.

    The two-sided probability ordering includes every fixed-margin table whose
    conditional probability is less than or equal to that of the observed table.
    """
    total_n = sum(row_totals)
    total_success = sum(successes)
    log_den = logcomb(total_n, total_success)
    log_p_obs = sum(logcomb(n, x) for n, x in zip(row_totals, successes)) - log_den
    p_total = 0.0
```

```python
    def enumerate_tables(i: int, remaining: int, log_num: float) -> None:
        nonlocal p_total
        if i == len(row_totals) - 1:
            x = remaining
            if 0 <= x <= row_totals[i]:
                lp = log_num + logcomb(row_totals[i], x) - log_den
                if lp <= log_p_obs + tol:
                    p_total += math.exp(lp)
            return
        remaining_capacity = sum(row_totals[i + 1:])
        lo = max(0, remaining - remaining_capacity)
        hi = min(row_totals[i], remaining)
        for x in range(lo, hi + 1):
            enumerate_tables(i + 1, remaining - x, log_num + logcomb(row_totals[i], x))

    enumerate_tables(0, total_success, 0.0)
    return min(1.0, p_total)


def rate_text(k: int, n: int) -> str:
    return f"{100 * k / n:.1f}%"


def main() -> None:
    parser = argparse.ArgumentParser()
    parser.add_argument("--input", required=True, help="Authoritative row-level analytic CSV.")
    parser.add_argument("--outdir", required=True, help="Directory for generated public package files.")
    args = parser.parse_args()

    inp = Path(args.input)
    outdir = Path(args.outdir)
    outputs = outdir / "outputs"
    outputs.mkdir(parents=True, exist_ok=True)

    df = pd.read_csv(inp, dtype=str, keep_default_na=False, encoding="utf-8-sig")
    required = [f"Q{i}" for i in range(1, 11)]
    missing_columns = [c for c in required if c not in df.columns]
    if missing_columns:
        raise ValueError(f"Missing required columns: {missing_columns}")

    n = len(df)
    if n != 393:
        raise ValueError(f"Expected 393 retained records; found {n}.")

    q4_rec = (df["Q4"] != "") & (df["Q4"] != Q4_NONREC)
    q5_rec = (df["Q5"] != "") & (df["Q5"] != Q5_NONREC)
    q6_unc = df["Q6"] == UNCERTAIN
    q6_eng = ~q6_unc
    q7_any = (df["Q7"] != "") & (df["Q7"] != Q7_NO)
    q7_no = df["Q7"] == Q7_NO
    q8_any = (df["Q8"] != "") & (df["Q8"] != Q8_OBS)
    q8_obs = df["Q8"] == Q8_OBS
    q9_drink = df["Q9"].str.startswith("Drink Me")
    q10_yes = (df["Q10"] != "") & (df["Q10"] != Q10_NO)
    q10_no = df["Q10"] == Q10_NO

    recognition_profile = (~q4_rec) & (~q5_rec) & ~(q6_unc & q7_no & q8_obs)
    engagement_profile = (q6_unc & q7_no & q8_obs) & (q4_rec | q5_rec)
    complete_profile = (~q4_rec) & (~q5_rec) & q6_unc & q7_no & q8_obs

    # Summary counts and proportions
    summary = [
        ("Q6 Engaged (responses 1–5)", int(q6_eng.sum()), n),
        ('Q6 Uncertain ("I’m not sure")', int(q6_unc.sum()), n),
        ("Q4 Thematic recognition", int(q4_rec.sum()), n),
        ("Q5 Affective recognition", int(q5_rec.sum()), n),
        ("Q7 No connection endpoint", int(q7_no.sum()), n),
        ("Q8 Observed-only endpoint", int(q8_obs.sum()), n),
        ("Q9 Drink Me preference", int(q9_drink.sum()), n),
        ("Q10 Structural-entry Yes", int(q10_yes.sum()), n),
        ("Q10 Structural-entry No", int(q10_no.sum()), n),
        ("Table 8 recognition-domain endpoint profile", int(recognition_profile.sum()), n),
        ("Table 8 engagement-domain endpoint profile", int(engagement_profile.sum()), n),
        ("Table 8 complete endpoint profile", int(complete_profile.sum()), n),
    ]
    pd.DataFrame(summary, columns=["measure", "count", "denominator"]).to_csv(
        outputs / "summary_counts.csv", index=False
    )
    prop_rows = []
    for label, k, den in summary:
        lo, hi = wilson_ci(k, den)
        prop_rows.append([label, k, den, k / den, lo, hi])
    pd.DataFrame(
        prop_rows,
        columns=["measure", "count", "denominator", "proportion", "wilson_95ci_low", "wilson_95ci_high"],
    ).to_csv(outputs / "summary_proportions.csv", index=False)

    # Q1–Q3 × Q6 crosstabs
    ctab_rows = []
    chi_rows = []
```

```python
for var, levels in [("Q1", Q1_LEVELS), ("Q2", Q2_LEVELS), ("Q3", Q3_LEVELS)]:
    table = []
    for level in levels:
        mask = df[var] == level
        engaged = int((mask & q6_eng).sum())
        uncertain = int((mask & q6_unc).sum())
        total = engaged + uncertain
        table.append([engaged, uncertain])
        ctab_rows.append([var, level, engaged, uncertain, total, engaged / total])
    arr = np.asarray(table, dtype=int)
    chi2, dof, p, v = chi2_cramers_v(arr)
    chi_rows.append([f"{var} × Q6", "Pearson chi-square", chi2, dof, p, v, "Cramér's V"])
pd.DataFrame(
    ctab_rows,
    columns=["variable", "category", "engaged", "uncertain", "total", "engagement_rate"],
).to_csv(outputs / "q1_q3_by_q6_crosstabs.csv", index=False)
pd.DataFrame(
    chi_rows,
    columns=["test", "test_type", "statistic", "df", "p_value", "effect_size", "effect_label"],
).to_csv(outputs / "q1_q3_by_q6_tests.csv", index=False)

# Collapsed age Fisher test
under = {"11 and younger", "12–14", "15–17", "18–24"}
older = {"25–34", "35–44", "45–54", "55 and older"}
under_mask = df["Q1"].isin(under)
older_mask = df["Q1"].isin(older)
age_table = [
    [int((under_mask & q6_eng).sum()), int((under_mask & q6_unc).sum())],
    [int((older_mask & q6_eng).sum()), int((older_mask & q6_unc).sum())],
]
age_p = stats.fisher_exact(age_table, alternative="two-sided").pvalue
pd.DataFrame(
    [
        ["Under 25", age_table[0][0], age_table[0][1], sum(age_table[0]), age_p],
        ["25 and older", age_table[1][0], age_table[1][1], sum(age_table[1]), age_p],
    ],
    columns=["age_group", "engaged", "uncertain", "total", "fisher_two_sided_p"],
).to_csv(outputs / "collapsed_age_fisher.csv", index=False)

# Six Q10 associations, RR, CIs, Fisher, BH-FDR
outcomes = [
    ("Q4: Thematic recognition", q4_rec),
    ("Q5: Affective recognition", q5_rec),
    ("Q6: Uncertainty", q6_unc),
    ("Q7: Personal resonance (any)", q7_any),
    ("Q8: Active meaning-making (any)", q8_any),
    ('Q9: Preference = "Drink Me"', q9_drink),
]
q10_rows = []
q10_ps = []
for label, outcome in outcomes:
    a = int((q10_no & outcome).sum())
    b = int((q10_no & ~outcome).sum())
    c = int((q10_yes & outcome).sum())
    d = int((q10_yes & ~outcome).sum())
    rr, lo, hi, corrected = rr_log_ci(a, b, c, d)
    p = stats.fisher_exact([[a, b], [c, d]], alternative="two-sided").pvalue
    q10_ps.append(p)
    q10_rows.append({
        "outcome": label,
        "q10_no_n": a,
        "q10_no_N": a + b,
        "q10_no_percent": 100 * a / (a + b),
        "q10_yes_n": c,
        "q10_yes_N": c + d,
        "q10_yes_percent": 100 * c / (c + d),
        "risk_ratio_yes_vs_no": rr,
        "rr_95ci_low": lo,
        "rr_95ci_high": hi,
        "rr_uses_0.5_correction": corrected,
        "fisher_two_sided_p": p,
    })
q10_q = multipletests(q10_ps, method="fdr_bh")[1]
for row, q in zip(q10_rows, q10_q):
    row["bh_fdr_q_across_6"] = q
q10_df = pd.DataFrame(q10_rows)
q10_df.to_csv(outputs / "q10_q4_q9_associations.csv", index=False)
q10_df.to_csv(outputs / "table9.csv", index=False)

# Q2 × Q10/Q6/Q8/Q9 Fisher-Freeman-Halton tests and BH-FDR
row_totals = [int((df["Q2"] == level).sum()) for level in Q2_LEVELS]
q2_outcomes = [
    ("Q10 structural-entry reports", q10_yes),
    ("Q6 uncertainty", q6_unc),
    ("Q8 active meaning-making", q8_any),
    ("Q9 Drink Me preference", q9_drink),
]
q2_rows = []
q2_ps = []
for label, outcome in q2_outcomes:
    successes = [int(((df["Q2"] == level) & outcome).sum()) for level in Q2_LEVELS]
```

```
        p = fisher_freeman_halton_exact(row_totals, successes)
        q2_ps.append(p)
        rates = [100 * k / den for k, den in zip(successes, row_totals)]
        q2_rows.append({
            "outcome": label,
            "no_experience_n": successes[0],
            "no_experience_N": row_totals[0],
            "no_experience_percent": rates[0],
            "beginner_n": successes[1],
            "beginner_N": row_totals[1],
            "beginner_percent": rates[1],
            "intermediate_n": successes[2],
            "intermediate_N": row_totals[2],
            "intermediate_percent": rates[2],
            "advanced_n": successes[3],
            "advanced_N": row_totals[3],
            "advanced_percent": rates[3],
            "professional_n": successes[4],
            "professional_N": row_totals[4],
            "professional_percent": rates[4],
            "fisher_freeman_halton_two_sided_p": p,
        })
    q2_q = multipletests(q2_ps, method="fdr_bh")[1]
    interpretations = [
        "No simple expertise gradient",
        "Mixed, non-monotonic pattern",
        "Not significant after BH-FDR",
        "No simple expertise gradient",
    ]
    for row, q, interp in zip(q2_rows, q2_q, interpretations):
        row["bh_fdr_q_across_4"] = q
        row["interpretation"] = interp
    q2_df = pd.DataFrame(q2_rows)
    q2_df.to_csv(outputs / "q2_sensitivity_ffh.csv", index=False)
    q2_df.to_csv(outputs / "table10.csv", index=False)

    # Consolidated tests file
    tests = []
    tests.append({
        "analysis_family": "Age sensitivity",
        "test": "Collapsed under-25 versus 25-and-older × Q6",
        "test_type": "Fisher exact (two-sided)",
        "p_value": age_p,
        "bh_fdr_q": "",
    })
    for row in q10_rows:
        tests.append({
            "analysis_family": "Q10 associations",
            "test": row["outcome"],
            "test_type": "Fisher exact (two-sided)",
            "p_value": row["fisher_two_sided_p"],
            "bh_fdr_q": row["bh_fdr_q_across_6"],
        })
    for row in q2_rows:
        tests.append({
            "analysis_family": "Q2 sensitivity",
            "test": row["outcome"],
            "test_type": "Fisher-Freeman-Halton exact (two-sided)",
            "p_value": row["fisher_freeman_halton_two_sided_p"],
            "bh_fdr_q": row["bh_fdr_q_across_4"],
        })
    pd.DataFrame(tests).to_csv(outputs / "tests.csv", index=False)

    effects = q10_df[
        ["outcome", "risk_ratio_yes_vs_no", "rr_95ci_low", "rr_95ci_high", "rr_uses_0.5_correction"]
    ].copy()
    effects.to_csv(outputs / "risk_ratios_and_95CIs.csv", index=False)

    # Missingness
    miss = []
    for item in [f"Q{i}" for i in range(1, 11)]:
        answered = int((df[item] != "").sum())
        missing = n - answered
        miss.append([item, answered, missing, 100 * missing / n])
    pd.DataFrame(
        miss, columns=["item", "answered_n", "missing_n", "missing_percent"]
    ).to_csv(outdir / "missingness_by_item.csv", index=False)

    # Codebook
    codebook = [
        ["Q1_age_group", "Age group", "categorical", "; ".join(Q1_LEVELS)],
        ["Q1_collapsed", "Collapsed age sensitivity grouping", "categorical",
         "Under 25 = Q1 categories through 18–24; 25 and older = Q1 categories 25–34 through 55 and older; undisclosed age
excluded"],
        ["Q2_dance_experience", "Dance/performing-arts experience", "categorical", "; ".join(Q2_LEVELS)],
        ["Q3_familiarity_abstraction", "Familiarity with abstract/unconventional performance", "categorical", ";
".join(Q3_LEVELS)],
        ["Q4_recognized", "Thematic recognition", "binary",
         "1 = any substantive option, including Other; 0 = explicit non-recognition as sole endorsement"],
        ["Q5_recognized", "Affective recognition", "binary",
         "1 = any substantive option, including Other; 0 = explicit non-recognition as sole endorsement"],
```

```
        ["Q6_uncertain", "Uncertainty endpoint", "binary",
         '1 = "I’m not sure."; 0 = Q6 responses 1-5'],
        ["Q7_resonance_any", "Personal resonance present", "binary",
         "1 = Q7 responses 1–4; 0 = no-connection endpoint"],
        ["Q8_active_any", "Active meaning-making present", "binary",
         "1 = Q8 responses 1–4; 0 = observed-only endpoint"],
        ["Q9_drink_me", "Internal-contrast preference", "binary",
         "1 = Drink Me; 0 = any EtoM-governed section"],
        ["Q10_structural_entry", "Structural-entry report", "binary",
         "1 = at least one listed structural descriptor endorsed; 0 = only explicit non-resonance option"],
        ["RR_Q10", "Risk ratio for Q10 association", "continuous",
         "Outcome risk in Q10 Yes divided by outcome risk in Q10 No; 0.5 added to all four cells only when a zero cell occurs"],
        ["BH_Q10", "BH-FDR adjusted value across six Q10 tests", "continuous", "Benjamini–Hochberg correction across Q10 × Q4–
Q9"],
        ["FFH_Q2", "Q2 sensitivity exact test", "continuous", "Two-sided Fisher–Freeman–Halton exact test for each 5×2 table"],
        ["BH_Q2", "BH-FDR adjusted value across four Q2 tests", "continuous", "Benjamini–Hochberg correction across Q2 ×
Q10/Q6/Q8/Q9"],
    ]
    pd.DataFrame(
        codebook, columns=["variable", "description", "type", "levels_or_coding"]
    ).to_csv(outdir / "codebook.csv", index=False)

    # QA alignment against final manuscript
    qa = []
    def check(name: str, observed: float, expected: float, tolerance: float = 1e-10) -> None:
        passed = abs(float(observed) - float(expected)) <= tolerance
        qa.append([name, observed, expected, tolerance, "PASS" if passed else "FAIL"])

    check("Analytic N", n, 393, 0)
    check("Table 2 Q6 engaged count", q6_eng.sum(), 362, 0)
    check("Table 2 Q6 uncertain count", q6_unc.sum(), 31, 0)
    check("Table 6 Q4 recognition count", q4_rec.sum(), 372, 0)
    check("Table 6 Q5 recognition count", q5_rec.sum(), 387, 0)
    check("Table 7 Q7 no-connection count", q7_no.sum(), 43, 0)
    check("Table 7 Q8 observed-only count", q8_obs.sum(), 78, 0)
    check("Table 8 recognition-domain endpoint count", recognition_profile.sum(), 0, 0)
    check("Table 8 engagement-domain endpoint count", engagement_profile.sum(), 9, 0)
    check("Table 8 complete endpoint count", complete_profile.sum(), 1, 0)
    check("Collapsed age Fisher p", age_p, 0.8176067429, 5e-11)

    manuscript_q10_p = [3.7409498040877824e-27, 0.008321315203370079, 0.0016638155086252356, 2.948620040527483e-05,
3.282597906820172e-08, 6.873119232675497e-11]
    manuscript_q10_q = [2.2445698824526694e-26, 0.008321315203370079, 0.0019965786103502827, 4.4229300607912244e-05,
6.565195813640344e-08, 2.061935769802649e-10]
    expected_cells = [(11,361),(29,358),(8,23),(20,330),(12,303),(13,8)]
    expected_rr = [2.87,1.09,0.25,1.46,2.24,0.05]
    for i, row in enumerate(q10_rows):
        check(f"Table 9 row {i+1} Q10 No numerator", row["q10_no_n"], expected_cells[i][0], 0)
        check(f"Table 9 row {i+1} Q10 Yes numerator", row["q10_yes_n"], expected_cells[i][1], 0)
        check(f"Table 9 row {i+1} RR rounded to 2 decimals", round(row["risk_ratio_yes_vs_no"],2), expected_rr[i], 0)
        check(f"Table 9 row {i+1} Fisher exact p", row["fisher_two_sided_p"], manuscript_q10_p[i], 1e-14)
        check(f"Table 9 row {i+1} BH-FDR q", row["bh_fdr_q_across_6"], manuscript_q10_q[i], 1e-14)

    manuscript_q2_p = [0.5841, 0.0052, 0.1036, 0.9697]
    manuscript_q2_q = [0.7788, 0.0209, 0.2073, 0.9697]
    for i, row in enumerate(q2_rows):
        check(f"Table 10 row {i+1} FFH p manuscript rounding", round(row["fisher_freeman_halton_two_sided_p"],4),
manuscript_q2_p[i], 0)
        check(f"Table 10 row {i+1} BH-FDR q manuscript rounding", round(row["bh_fdr_q_across_4"],4), manuscript_q2_q[i], 0)

    qa_df = pd.DataFrame(
        qa, columns=["check", "observed", "expected", "tolerance", "result"]
    )
    qa_df.to_csv(outdir / "qa_alignment_check.csv", index=False)
    if (qa_df["result"] != "PASS").any():
        failed = qa_df.loc[qa_df["result"] != "PASS", "check"].tolist()
        raise RuntimeError(f"QA alignment failed: {failed}")


if __name__ == "__main__":
    main()
```

scripts/requirements.txt

```
numpy>=1.26,<3
pandas>=2.1,<3
scipy>=1.11,<2
statsmodels>=0.14,<1
```
